# Hierarchical Quatsome-RGD Nanoarchitectonic Surfaces for Enhanced Integrin-Mediated Cell Adhesion

**DOI:** 10.1021/acsami.2c10497

**Published:** 2022-10-17

**Authors:** Marc Martínez-Miguel, Miquel Castellote-Borrell, Mariana Köber, Adriana R. Kyvik, Judit Tomsen-Melero, Guillem Vargas-Nadal, Jose Muñoz, Daniel Pulido, Edgar Cristóbal-Lecina, Solène Passemard, Miriam Royo, Marta Mas-Torrent, Jaume Veciana, Marina I. Giannotti, Judith Guasch, Nora Ventosa, Imma Ratera

**Affiliations:** † Institut de Ciència de Materials de Barcelona (ICMAB-CSIC), Campus UAB, Bellaterra 08193, Spain; ‡ Biomedical Research Networking Center on Bioengineering, Biomaterials and Nanomedicine (CIBER-BBN), Madrid 28029, Spain; § Institut de Química Avançada de Catalunya (IQAC−CSIC), Barcelona 08034, Spain; ∥ Nanoprobes and Nanoswitches group, Institute for Bioengineering of Catalonia (IBEC), The Barcelona Institute of Science and Technology (BIST), Barcelona 08028, Spain; ⊥ Departament de Ciència dels Materials i Química Física, Universitat de Barcelona, Barcelona 08028, Spain; # Dynamic Biomimetics for Cancer Immunotherapy, Max Planck Partner Group, ICMAB-CSIC, Campus UAB, Bellaterra 08193, Spain; ∇ Unidad de Péptidos, UB, Unidad asociada al CSIC por el IQAC, Barcelona 08028, Spain

**Keywords:** nanovesicles, quatsomes, self-assembled monolayers, Arg-Gly-Asp (RGD), cell adhesion, tissue engineering, integrins, surface engineering

## Abstract

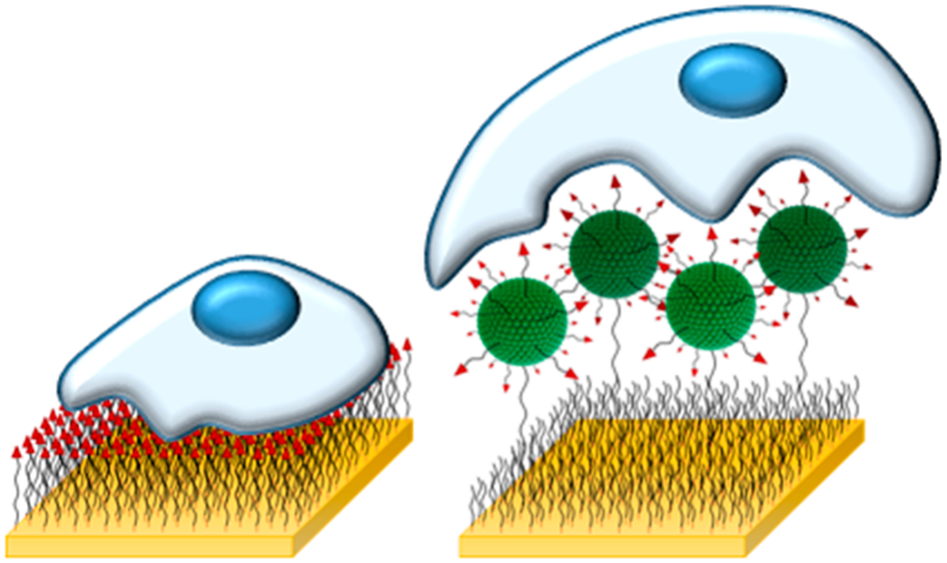

The synthesis and study of the tripeptide Arg-Gly-Asp (RGD), the binding site of different extracellular matrix proteins, e.g., fibronectin and vitronectin, has allowed the production of a wide range of cell adhesive surfaces. Although the surface density and spacing of the RGD peptide at the nanoscale have already shown a significant influence on cell adhesion, the impact of its hierarchical nanostructure is still rather unexplored. Accordingly, a versatile colloidal system named quatsomes, based on fluid nanovesicles formed by the self-assembling of cholesterol and surfactant molecules, has been devised as a novel template to achieve hierarchical nanostructures of the RGD peptide. To this end, RGD was anchored on the vesicle’s fluid membrane of quatsomes, and the RGD-functionalized nanovesicles were covalently anchored to planar gold surfaces, forming a state of quasi-suspension, through a long poly(ethylene glycol) (PEG) chain with a thiol termination. An underlying self-assembled monolayer (SAM) of a shorter PEG was introduced for vesicle stabilization and to avoid unspecific cell adhesion. In comparison with substrates featuring a homogeneous distribution of RGD peptides, the resulting hierarchical nanoarchitectonic dramatically enhanced cell adhesion, despite lower overall RGD molecules on the surface. The new versatile platform was thoroughly characterized using a multitechnique approach, proving its enhanced performance. These findings open new methods for the hierarchical immobilization of biomolecules on surfaces using quatsomes as a robust and novel tissue engineering strategy.

## Introduction

The heterologous replacement of damaged organs and tissues is nowadays a well-established therapeutic approach. However, there are certain important limitations to this procedure, such as immunological incompatibility and the shortage of organ donors.^1^ Tissue engineering, through the combination of the principles of material engineering and life sciences, aims at presenting a solution to these constraints.^2^ The most recent tissue engineering strategies rely on the combination of cells and adequate growth factors with a scaffold that supports the tissue or organ.^3^ The choice of an adequate scaffold is of great importance due to its ability not only to physically support the tissue but also to direct the growth and position of cells^4−11^ and to tune other cellular functions, such as proliferation and differentiation.^12−14^ The design of the scaffolds is based on mimicking the natural extracellular matrix (ECM), which directly supports cell adhesion through specific interactions between its components and the cells.

Integrins are pivotal players which mediate cellular adhesion to surfaces.^15^ Their interaction with cell adhesion ligands triggers a response in the cell that starts with the recruitment of protein complexes to form subcellular structures called focal adhesions (FAs).^16^ FAs are not only signaling centers,^17^ but directly connect the inner cytoskeleton of the cell with the exterior, thus allowing the cell to physically sense the ECM or surface.^16,18,19^ Both the number and area of FAs are suitable parameters to study cell adhesion and migration through surfaces.^20^

The study of integrin-mediated cell adhesion on materials has been conducted both on complete ECM proteins, such as fibronectin and vitronectin, and on the minimum required peptide sequences that are integrin ligands, like the RGD peptide.^21,22^ The latter approach offers several advantages, such as the stability of peptides in comparison to entire proteins and the capacity to study the biological input in a simplified environment.^23^ RGD peptides have indeed been largely immobilized on surfaces in diverse configurations to study their effect on cell adhesion. For example, RGD-terminated self-assembled monolayers (SAMs) were prepared,^24,25^ which allow a homogeneous arrangement of the cell adhesion ligands on a surface while being able to tune the interfacial chemistry of the substrate. Other systems consisted of RGD-decorated polymer brushes, which are a semi-3D material that allows studying of integrin-dependent cell adhesion as a function of many parameters such as the substrate softness,^26^ the depth of the RGD motifs within the polymer scaffold,^27,28^ and the density of RGD ligands.^29^ Additionally, some studies on planar surfaces showed a clear impact on the density and spacing at the nanometer range of cell adhesion ligands.^30−33^ In addition, it has been reported that cells can respond to different nanostructured cell adhesion ligands,^34,35^ and the resulting FA structure is influenced by the underlying pattern. Altogether, the study of the factors that govern cell adhesion and their optimization is paramount to the development of appropriate and functional artificial scaffolds for tissue engineering.

Nanoarchitectonics is a novel concept that combines nanotechnology with other research disciplines like supramolecular chemistry. Specifically, self-assembly processes are crucial to describe this technology which can arrange nanosize structural units in advanced materials with a specific configuration. Nanoarchitectonics aims at opening a new paradigm of nanotechnology creating reliable nanomaterials or nanosystems by organizing nanoscale units where the main players are not the individual nano parts but their interactions, giving place to new functionalities.^36−40^ In this paper, we have used novel nanovesicles named quatsomes to develop RGD peptide nanoarchitectonic surfaces for enhanced integrin-mediated cell adhesion.

Quatsomes (QSs) are nonliposomal lipid-based unilamellar nanovesicles composed of self-assembled sterols and quaternary ammonium surfactants that present high morphological vesicle-to-vesicle homogeneity and stability.^41^ Importantly, QSs can be easily tuned with a wide range of chemical functionalities, making them promising nanocarriers for applications in nanomedicine.^42^ QSs have already been explored as nanocontainers to encapsulate drugs and protein cargos^43,44^ as well as fluorescent dyes for therapy and diagnostics.^45,46^ However, the integration of biomolecules on the fluid QSs membrane and their use once covalently anchored on surfaces are an unexplored nanoarchitectonic field.

**Figure 1:**
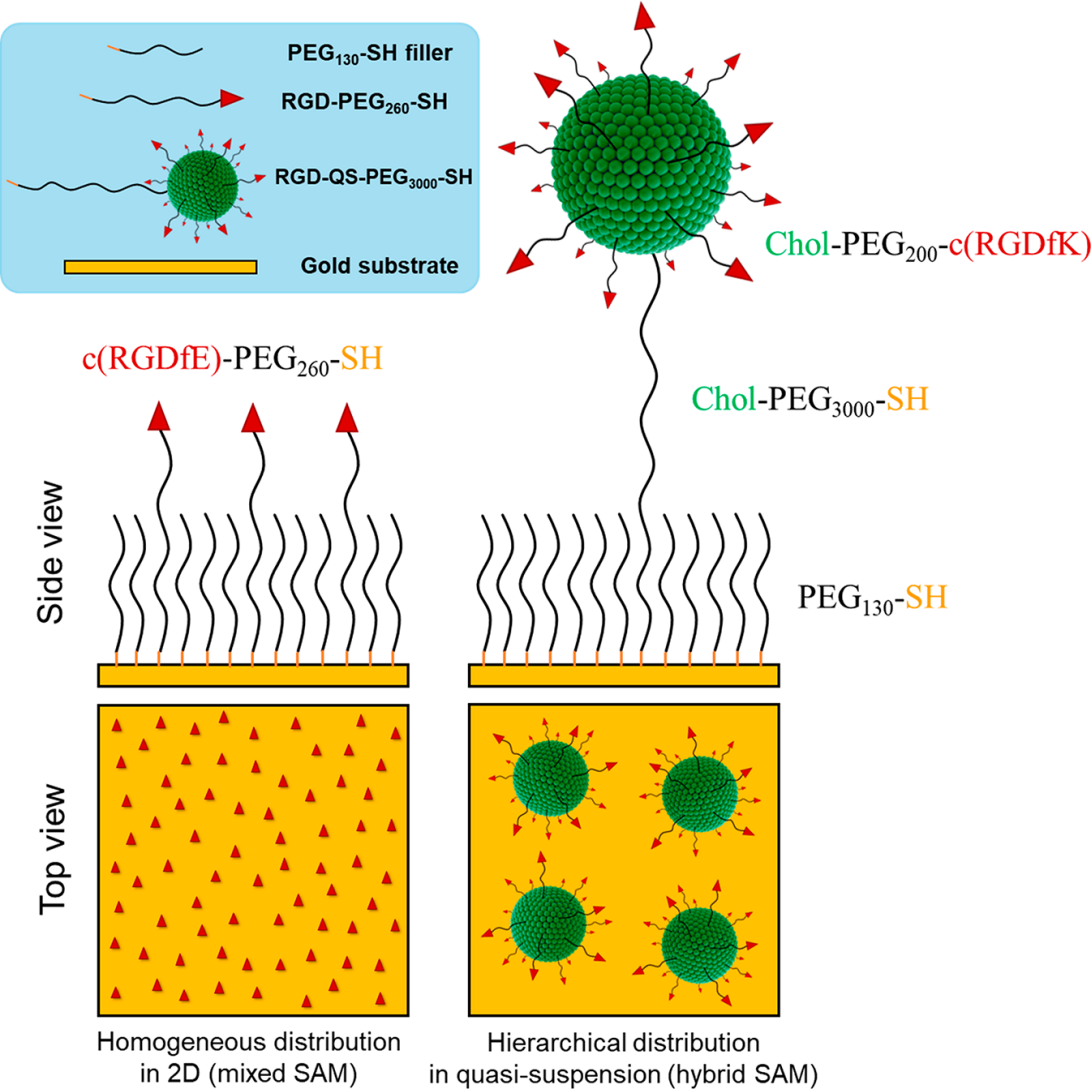
Schematic representation of the two kinds of substrates studied. On the left, a mixed SAM featuring thiolated PEG_130_-SH (PEG-filler) and thiolated RGD-terminated PEG (RGD-PEG_260_-SH). On the right, a hybrid SAM composed of thiolated PEG_130_-SH (PEG-filler) and thiol-functionalized RGD-quatsomes (RGD-QS-PEG_3000_-SH). Top view SAMs are depicted with the same bulk average surface RGD density. The *x* in PEG_*x*_ indicates the molecular weight of the PEG building block.

The conjugation of certain biomolecules on nanovesicle membranes can increase their activity, using an optimized orientation of their bioactive groups, as seen in liposomes conjugated with α-galactosidase A^47,48^ and in QSs conjugated with epidermal growth factor.^44^ Thus, QSs are herein presented as an effective nanoscopic building block to prepare hierarchically organized RGD surfaces for cell adhesion enhancement. For this goal, RGD-peptide-functionalized QS nanovesicles were synthesized, which comprise a few long poly(ethylene glycol) (PEG) chains terminated with thiol groups for gold grafting (RGD-QS-PEG_3000_-SH; see Figure 1 and Figure S1). Among the wide range of strategies for surface biofunctionalization, SAMs have been demonstrated to be valuable for engineering well-defined surfaces with tunable chemistries to study and control cell adhesion.^49,50^ Thus, the capabilities of the resulting RGD functionalized QSs to increase cell adhesion were assessed by covalently anchoring them on gold surfaces via SAMs, which provide an optimal environment for the hierarchical nanostructuration of the RGD peptide ligands exposed on the fluidic nanovesicle surface (see Figure 1).

The present approach consists of: (i) the production of suspensions of RGD-peptide-functionalized QS unilamellar nanovesicles (RGD-QS-PEG_3000_-SH, Figure 1) through a single-step procedure based on compressed CO_2_,^51^ and (ii) their subsequent covalent anchoring on gold surfaces through the long PEG thiol terminated chain, together with a PEG-filler (PEG_130_-SH; see Figure 1) to form hybrid SAMs. The RGD-QS-PEG_3000_-SH features a thiol group at the end of a long PEG chain, which interacts with gold to form a covalent bond. Due to this long PEG chain, QSs are found in a state of quasi-suspension: not completely immobilized but neither able to move away from the functionalized surface, facilitating the accessibility of RGD ligands to cells. In this work we demonstrate that the novel engineered multifunctional nanovesicles present: (1) good physicochemical properties, (2) the capability to be covalently anchored to gold surfaces (Figure 1), and (3) the ability to induce better cell adhesion than homogeneous RGD-terminated mixed SAMs based on thiolated PEG (PEG_130_-SH) and thiolated RGD-terminated PEG (RGD-PEG_260_-SH, Figure 1). The PEG/RGD-PEG mixed SAMs feature a concentration-dependent homogeneous distribution of RGD ligands on the 2D surface. Our results show that the covalent anchoring of the nanovesicles to a gold surface in such a state of quasi-suspension facilitates the accessibility of RGD ligands to cells. To deeply characterize this complex system, an extensive multitechnique characterization at the nanoscale is required to assess the integrity of the QS nanovesicles anchored on the surface for the first time, as well as the accessibility of the integrated RGD ligands to interact with integrins and their enhanced cell-adhesion capabilities. In general, the system presented here offers a novel strategy for the hierarchical nanostructuration of bioactive molecules of interest, not only for fundamental studies but also for direct applications in tissue engineering to immobilize the relevant biomolecules on surfaces or scaffolds.

## Results and Discussion

### Multifunctional Quatsome Production

Nanovesicle formulations of blank QS-PEG_3000_-SH (quatsomes without RGD functionalization) and RGD-QS-PEG_3000_-SH in water were produced using a CO_2_-based technology, named DELOS-susp, which enables a high control of the molecular self-assembly process and the preparation of formulations with large vesicle-to-vesicle homogeneity and low dispersity.^43,44,51,52^ The prepared nanovesicle formulations were further diafiltrated to ensure the separation of molecules not integrated into the quatsome membrane. These nanovesicles were composed of a mixture of cholesterol and cholesterol derivatives, and myristalkonium chloride (MKC), a quaternary ammonium surfactant that is the C14 homologue of the widely used benzalkonium chloride in pharmaceutical formulations. Besides cholesterol, two additional cholesterol derivatives were present. One of them was cholesterol bound to a cyclic RGD peptide through a poly(ethylene glycol) spacer (chol-PEG_200_-c(RGDfK)), which provides a ligand for integrins to the nanovesicles. This molecular building block was synthesized as previously described.^43,53^ The other one was a commercially available cholesterol molecule, functionalized with a long thiol-terminated poly(ethylene glycol) chain (chol-PEG_3000_-SH) with a contour length of ca. 21 nm,^54^ providing the nanovesicles with a flexible Au-anchoring moiety. The chemical structure of all the molecules employed for quatsome production can be found in Section 1 of the Figure S1.

The concentration of cholesterol-PEG_200_-c(RGDfK) and therefore the concentration of RGD peptide attached to the RGD-QS-PEG_3000_-SH nanovesicle formulation was measured using high-performance liquid chromatography (HPLC) coupled to an evaporative light scattering detector (ELSD) (see the Supporting Information, Section 4 II). These measurements revealed that approximately 5% of the total cholesterol in the formulation is cholesterol-PEG_200_-c(RGDfK). As detailed later, this value was used to estimate the bulk average surface RGD density and the local average surface RGD density of hybrid SAMs (see Table 1 and the Supporting Information, Section 4).

**Table 1:** Bulk and Local RGD Peptide Density of Mixed and Hybrid SAMs and Composition of the Incubation Formulations Used to Prepare Them

Platform	Sample	Incubation solution composition^a^ (mol %)	Incubation solution conc.^b^ (μM)	Estimated maximum bulk average surface RGD density^c^ (RGD units/nm^2^)	Estimated maximum local average surface RGD density^d^ (RGD units/nm^2^)
Mixed SAM RGD	SAM PEG 100%	100% PEG_130_-SH	1000	0	0
	SAM RGD 100%	100% RGD-PEG_260_-SH	1000	1.22	1.22
	SAM RGD 10%	10% RGD-PEG_260_-SH	100	0.122	0.122
		90% PEG_130_-SH	900		
	SAM RGD 1%	1% RGD-PEG_260_-SH	10	0.0122	0.0122
		99% PEG_130_-SH	990		
Hybrid SAM RGD-QS	SAM BLANK-QS 100%	100% QS-PEG_3000_-SH	0.022	0	0
	SAM RGD-QS 100%	100% RGD-QS-PEG_3000_-SH	0.022	0.078	0.078
	SAM RGD-QS 10%	10% RGD-QS-PEG_3000_-SH	0.022	∼0.0078–0.078	0.078
		90% PEG_130_-SH	0.2		
	SAM RGD-QS 1.5%	1.5% RGD-QS-PEG_3000_-SH	0.027	∼0.0012–0.078	0.078
		98.5% PEG_130_-SH	1.8		
	SAM RGD-QS 0.125%	0.125% RGD-QS-PEG_3000_-SH	0.025	∼0.0001–0.078	0.078
		99.875% PEG_130_-SH	19.8		

a Molar composition in %.

b Concentration of the components of the solutions in which surfaces were incubated to produce SAMs. QS is considered a supramolecular unit for the calculation of molar concentrations with an approximate molecular weight of 1.86 × 10^7^ ± 0.78 × 10^7^ Da (see the Supporting Information Section 3).

c Estimated by calculating the packing density of the RGD units on a flat surface.

d In mixed SAMs, RGD is not localized over QS nanovesicles, and therefore local and bulk density will be the same; in hybrid SAMs, the local density of RGD units is the same as the density of RGD units on the QS surface and will not change with QS dilution on the SAM.

### Physicochemical and Morphological Characterization of Multifunctional Quatsomes

**Figure 2:**
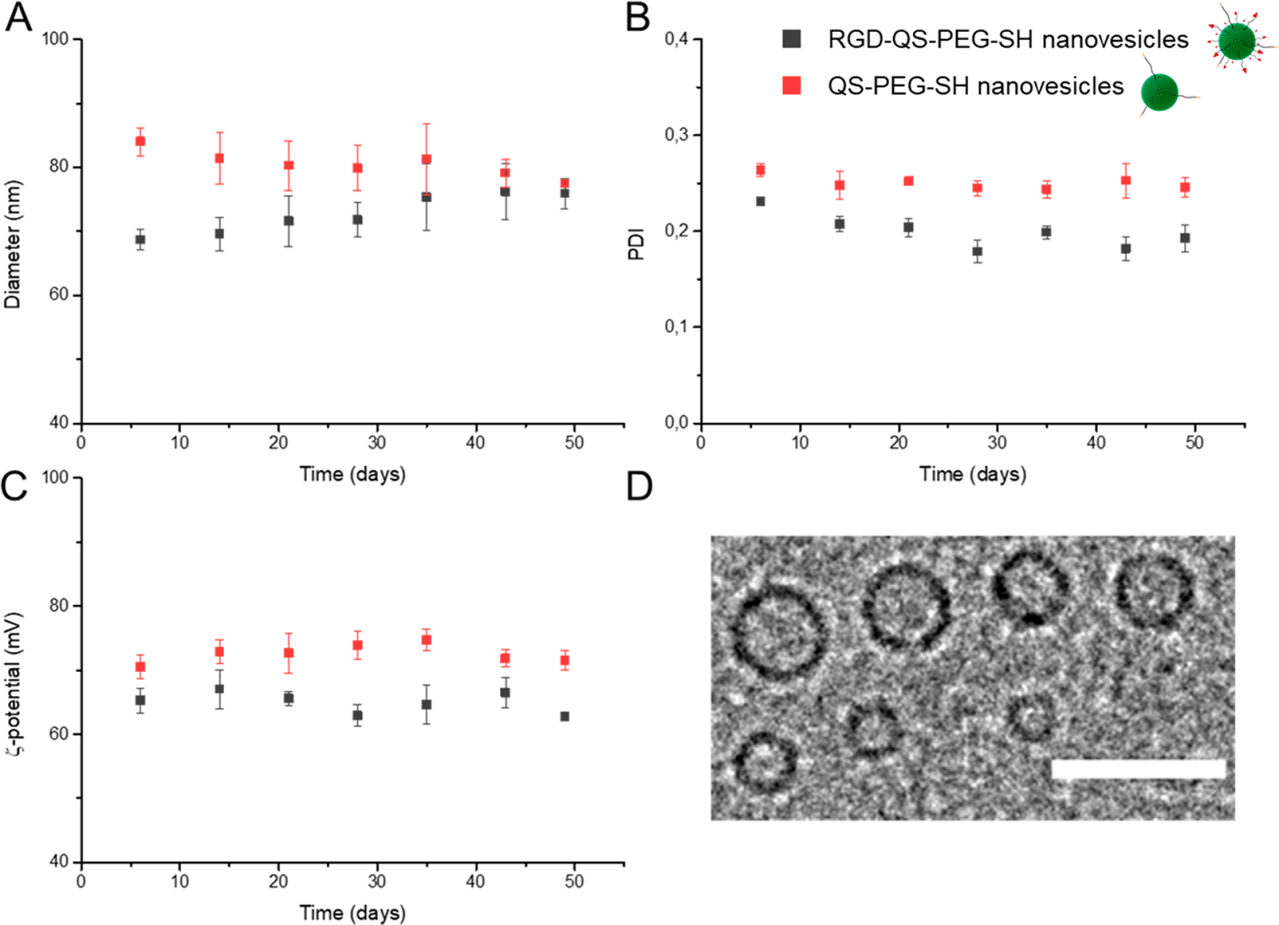
Long-term colloidal stability study of RGD-functionalized thiol-terminated quatsomes (i.e., the RGD-QS-PEG_3000_-SH and nonfunctionalized thiol-terminated quatsome QS-PEG_3000_-SH measured by DLS and electrophoretic light scattering (ELS) over 50 days. (A) Mean hydrodynamic diameter; (B) polydispersity index (PdI); and (C) apparent ζ-potential vs time. (D) Representative cryo-TEM image of thiol-terminated RGD-QS-PEG_3000_-SH 3 weeks after production. Scale bar = 100 nm.

Thiol-terminated RGD-QSs (RGD-QS-PEG_3000_-SH) have a size distribution, with a mean diameter *d̅* of around 70 nm and a low polydispersity index (PdI) of ∼0.2 (Figure 2A, B). It is important to note that intensity-weighed size distributions obtained from dynamic light scattering (DLS) are largely influenced by the presence of larger-sized vesicles, as the scattered light intensity is proportional to *d*^4^. The relatively high apparent ζ-potential of ∼65 mV results in the electrostatic repulsion of vesicles, facilitating vesicle stability over time. Importantly, no significant changes were observed in vesicle size and apparent ζ-potential over 50 days (Figure 2A–C). The thiol-terminated blank QSs without RGD (QS-PEG_3000_-SH) showed slightly higher values in mean size, dispersity index, and apparent ζ-potential (Figure 2) and were also stable for 50 days.

Multifunctional quatsomes were characterized by transmission electron microscopy under cryogenic conditions (cryo-TEM) 3 weeks after their production. The nanovesicles were unilamellar with spherical morphology, and with size in accordance with the DLS measurements (Figure 2D). Additionally, the membrane thickness was measured from the images, yielding a value of 5 nm. The high colloidal stability of these nanovesicles is very relevant to ensure the robustness and reproducibility of the hybrid SAM preparation.

### Preparation of Mixed and Hybrid SAMs Based on Quatsomes

**Figure 3:**
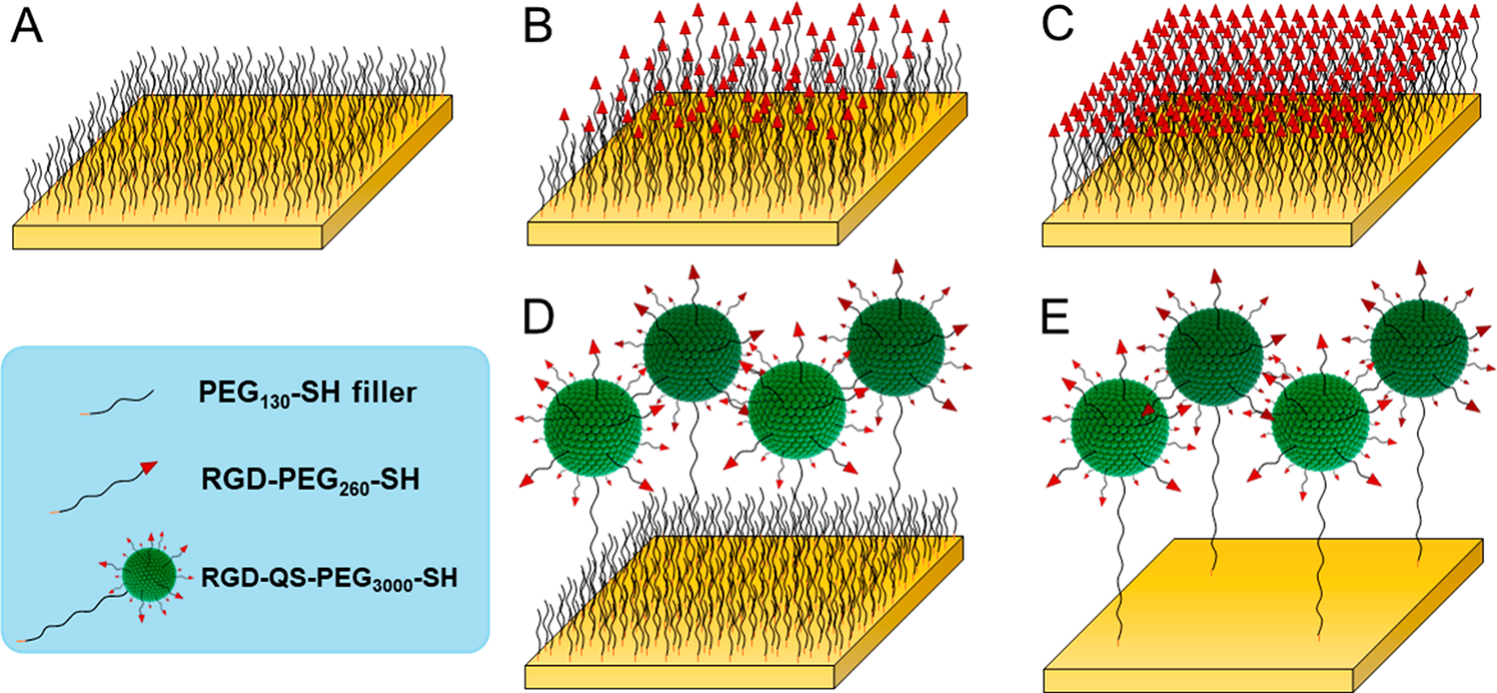
Schematic representations of the different types of SAMs produced. (A) Homogeneous SAM with 100% PEG_130_-SH filler (SAM PEG 100%); (B) mixed SAM (SAM RGD *x*%) with a mixture of RGD-PEG_260_-SH and PEG_130_-SH filler with *x*% RGD-PEG_260_-SH; (C) homogeneous SAM with 100% RGD-PEG_260_-SH (SAM RGD 100%); (D) hybrid SAM (SAM RGD-QS *x*%) with a mixture of RGD-QS-PEG_3000_-SH and PEG_130_-SH filler with *x*% RGD-QS PEG_3000_-SH component; and (E) homogeneous SAM (SAM RGD-QS 100%) with 100% RGD-QS-PEG_3000_-SH. Note that in E, the QSs are not standing up (as depicted in the figure) but ruptured upon interacting with the bare gold (vide infra).

Two kinds of functionalized surfaces were engineered. The first ones, named mixed SAMs, were produced by the incubation of gold substrates in solutions of RGD-PEG_260_-SH and PEG_130_-SH filler at different molar percentages, hereafter named as SAM RGD *x*%, where *x*% is the mol % RGD-PEG_260_-SH in the solution. The second functionalized substrates are called hybrid SAMs and were produced similarly, by incubation of formulations of RGD-QS-PEG_3000_-SH nanovesicles and PEG_130_-SH filler, with varying molar percentages between both components, hereafter named SAM RGD-QS *x*% with *x*% being the mol % of the RGD-QS-PEG_3000_-SH nanovesicles in the incubation formulation (Figure 3). For the calculations of molar concentrations, QSs were considered as discrete supramolecular units with an *M*_W_ of 1.86 × 10^7^ ± 0.78 × 10^7^ Da (see Section 3 of the Supporting Information for the RGD-QS-PEG_3000_-SH *M*_W_ determination and for the calculation of the molar concentration of RGD-QS-PEG_3000_-SH nanovesicles in the formulations).

The anchoring to the gold substrate in both cases, RGD-PEG_260_-SH single molecules and RGD-QS-PEG_3000_-SH nanovesicles, was driven by the gold–thiol interaction. The mixed SAMs were used as a control to evaluate the impact of the hierarchical RGD-quatsome nanoarchitectonics (hybrid SAMs) on integrin-mediated cell adhesion. Table 1 contains the characteristics of the prepared surfaces and, among other parameters, the molar ratios of their components in the incubation formulation and the estimated average bulk and local surface RGD density (see the Supporting Information Section 4), where the average bulk density refers to the total RGD density taking into account the whole surface and the local density to the RGD density on the quatsome. It should be noted that the amount of RGD to which the gold surfaces were exposed was higher for mixed SAMs in comparison to hybrid SAMs. Additionally, the total concentration of RGD present in the hybrid SAMs is higher than the concentration of RGD that is exposed to the environment due to the fraction of the chol-PEG_200_-c(RGDfK) molecules located in the inner membrane of the quatsome vesicles (see Figures S1 and S2).

**Figure 4:**
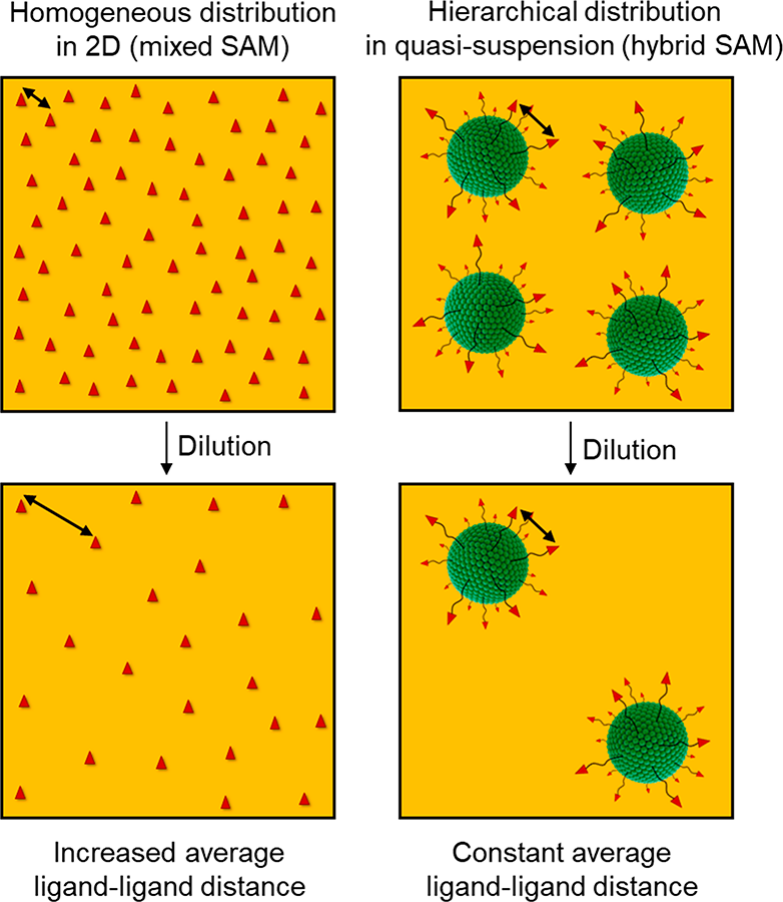
Schematic representation of the dilution of RGD ligands and its effect on the average RGD-to-RGD distance when homogeneously distributed on a surface (mixed SAM) vs when nanostructured on quatsomes  in quasi-suspension (hybrid SAM).

In the case of mixed SAMs, the lower the percentage of RGD-PEG_260_-SH in relation to PEG_130_-SH in the incubation solution, the larger the RGD-to-RGD average distance on the SAM (see Figure 4 and the Supporting Information, Section 5). Indeed, as the concentration of RGD-PEG_260_-SH in the incubation solution decreases, the bulk and local surface density of RGD-PEG_260_-SH molecules decrease too. By contrast, for hybrid SAMs, a reduction in the % of RGD-QS-PEG_3000_-SH in relation to PEG_130_-SH, does not impact the RGD-to-RGD distance on the surface. Even though the QS-to-QS distance increases due to the dilution process, the RGD-to-RGD distance is kept constant due to their localization over QS (see Figure 4). When producing these hybrid SAMs, it is important to note that two components of significantly different *M*_W_ are being combined in the same suspension (RGD-QS-PEG_3000_-SH with *M*_W_ = 1.86 × 10^7^ vs PEG_130_-SH with *M*_W_ = 294.45), and thus their incorporation rates to the gold surface may be very different. As shown in Table 1, the substrates featuring a homogeneous distribution of RGD are expected to always feature a bulk surface density of RGD at least 1 order of magnitude higher than that of the substrates where the quatsome vesicles are used to immobilize the RGD.

### Impact of RGD Hierarchical Nanoarchitectonic on Cell Adhesion

**Figure 5:**
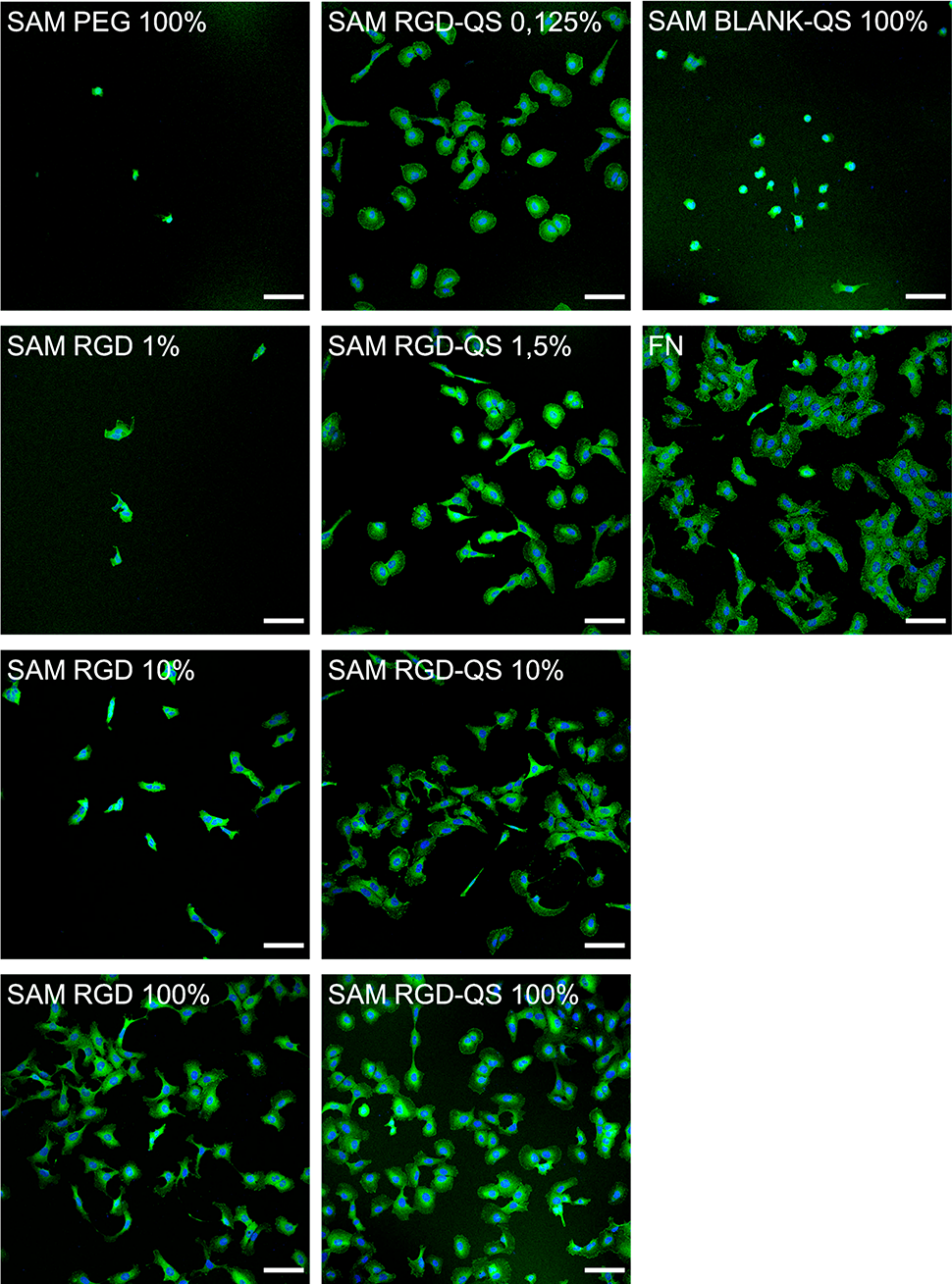
Representative confocal fluorescence microscopy images of U2OS cells (nuclei in blue, FA in green) seeded on the substrates (*N*_substrates_ = 10) detailed in Table 1. Scale bars = 100 μm.

**Figure 6:**
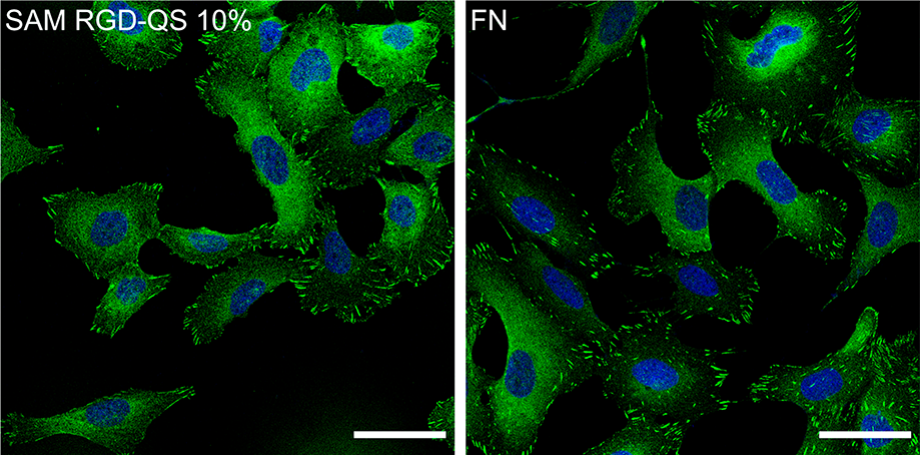
Representative confocal fluorescence microscopy images of U2OS cells seeded on SAM RGD-QS 10% (left) and FN (right) (nuclei in blue and FA in green). FA areas can be observed in the membrane as higher contrasted green areas. Scale bars = 100 μm.

**Figure 7:**
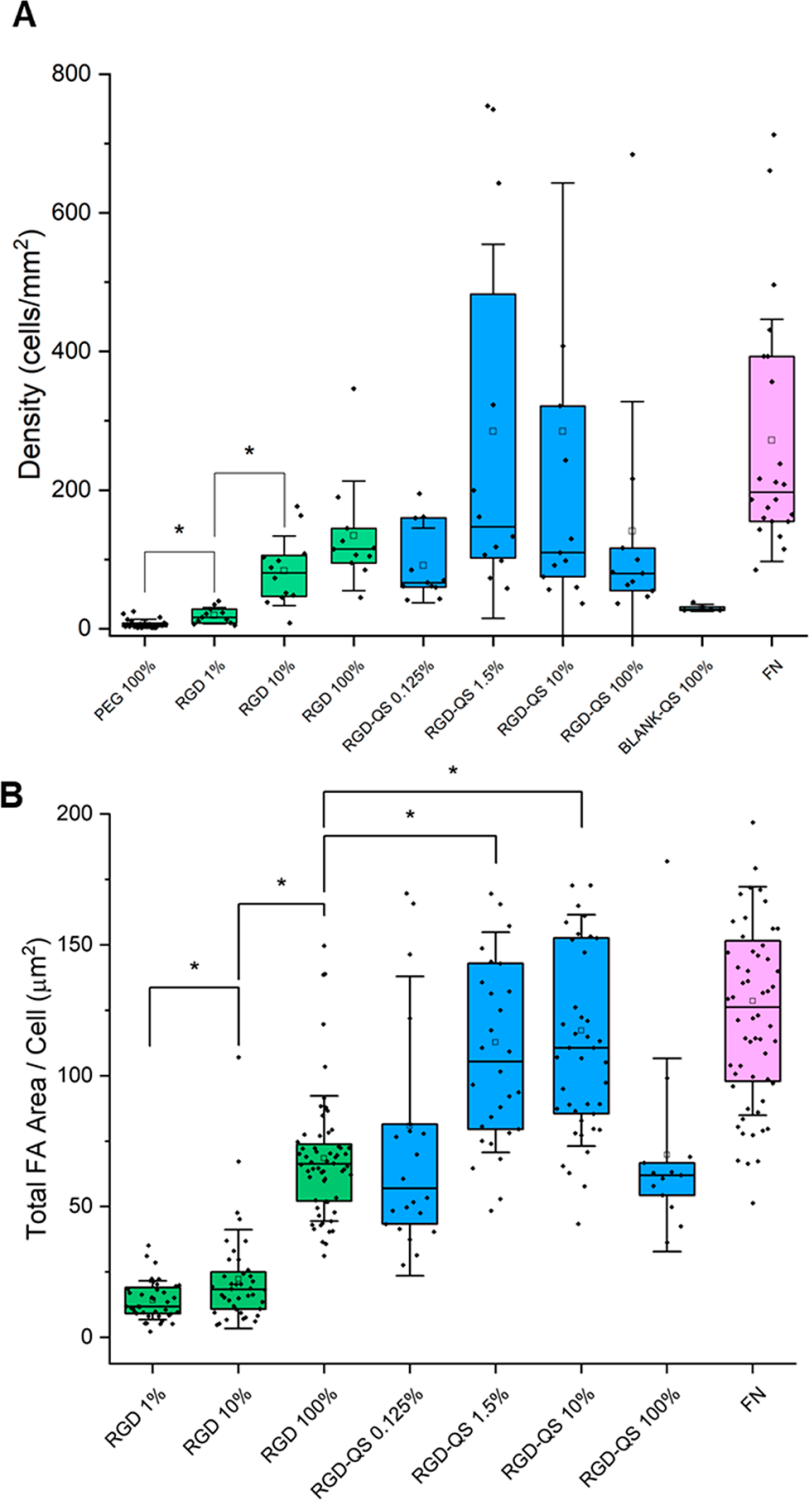
(A) Cell density and (B) total FA area per cell of human osteosarcoma (U2OS) cells seeded on two types of SAM surfaces: RGD-terminated mixed SAMs (SAM RGD *x*%) and hybrid SAMs (SAM RGD-QS *x*%). Both types of surfaces featured different molar ratios of RGD peptide and PEG-SH filler molecules, as indicated on the horizontal axes. SAM PEG 100% is a negative control. FN corresponds to fibronectin-coated gold, which is the positive control in which cell adhesion is expected to be maximized. Statistical analysis was performed with the Kruskal–Wallis ANOVA test (*p < 0.05).

Human U2OS osteosarcoma cells were seeded on top of the different surfaces depicted in Figure 3. The U2OS cell line was chosen due to its adherent properties and its suitability for immunofluorescence studies. A coating of fibronectin (FN) on gold was used as a positive control. Twenty-four hours after seeding, cells were fixed, immunostained, and imaged under a confocal fluorescence microscope (Figures 5 and 6). The median cell density and the median focal adhesion (FA) area per cell were calculated (Figure 7), as representative parameters to quantify the extension of integrin-mediated cell adhesion over the different surfaces.

The overall abundance and morphology of cells on the different substrates were correlated with cell adhesion on RGD-presenting and non-RGD-presenting samples. Cells seeded on the PEG and blank-QS 100% SAMs showed rounded morphologies, as no significant adherence to the substrates was observed, resulting in low cell viability. In contrast, cells seeded on the RGD-terminated and RGD-QS SAMs showed elongated morphologies, in agreement with their interaction with the underlying substrate and suggesting high cell viability.

As expected, cells were not able to adhere to SAMs of only PEG_130_-SH filler molecules (SAM PEG 100%), with a median of 6 cells/mm^2^ and no visible FAs, given the hydrophilic nature of this layer that impairs cell adhesion. Therefore, the cell surface density and the total FA area per cell are null (Figures 5 and 7). No significant adhesion was observed on the blank-QS SAMs either, with a median of 28 cells/mm^2^ and no visible FAs, due to the absence of adhesive motifs. When increasing the concentrations of RGD peptide on the mixed SAMs, SAM RGD *x*% (where *x* = 1, 10, and 100%), an increase in cell adhesion was observed, with medians of 17 cells/mm^2^ for SAM RGD 1%, 81 cells/mm^2^ for SAM RGD 10%, and 115 cells/mm^2^ for SAM RGD 100%. The surface with 100% RGD, exhibiting the maximum cell adhesion, was used as a control to evaluate the effect of the RGD hierarchic nanoarchitectonic promoted by the attachment of RGD to nanovesicles further anchored to the gold substrate (Figures 5 and 7 and Table 2).

**Table 2:** Summary of the Cell Adhesion Results for the Studied Surfaces

Sample	Estimated bulk average surface RGD density (RGD units/nm^2^)^a^	Estimated maximum local average surface RGD density ^E^ (RGD units/nm^2^)^b^	Median cell density (cells/mm^2^)^c^	Median total FA area/cell (μm^2^)^d^
SAM PEG 100%	0	0	6	-
SAM RGD 100%	1.22	1.22	115	66
SAM RGD 10%	0.122	0.122	81	18
SAM RGD 1%	0.0122	0.0122	17	12
SAM BLANK-QS 100%	0	0		
SAM RGD-QS 100%	0.078	0.078	80	62
SAM RGD-QS 10%	∼0.0078–0.078	0.078	110	111
SAM RGD-QS 1.5%	∼0.0012–0.078	0.078	147	105
SAM RGD-QS 0.125%	∼0.0001–0.078	0.078	67	57

a Estimated by calculating the packing density of the RGD units on a flat surface.

b In mixed SAMs, RGD is not localized over QS nanovesicles, and therefore local and bulk density are the same. In hybrid SAMs, the local density of RGD units is the same as the density of RGD units on the QS surface and does not change with QS dilution on the SAM.

c The median number of cells per area is seen in Figure 7A.

d The median total focal adhesion area per cell is seen in Figure 7B.

For the QS-based hybrid SAMs (SAM RGD-QS *x*%), we tested four molar ratios of RGD-QS-PEG_3000_-SH vs PEG_130_-SH filler molecules, namely, *x* = 0.125, 1.5, 10, and 100. Cell density values for these surfaces yielded medians of 67, 147, 110, and 80 cells/mm^2^ for SAM RGD-QS 0.125, 1.5, 10, and 100%, respectively (Figure 7A and Table 2). All these conditions yielded cell density parameters that are comparable to the SAM RGD 100% even when the surfaces featured 2 orders of magnitude less RGD overall density (see Table 1 and 2 and the Supporting Information for RGD density calculations). Regarding the total FA area per cell, mixed SAMs of increasing RGD content showed increasing cell adhesion on the surface, with medians of 12 μm^2^ for RGD 1%, 18 μm^2^ for RGD 10%, and 66 μm^2^ for RGD 100%. The total FA area per cell obtained for the hybrid SAMs with 0.125 and 100% RGD-QSs, with medians of 57 and 62 μm^2^, respectively, are not significantly different from that obtained with the SAM RGD 100%. Moreover, cells seeded on hybrid SAMs with 1.5 and 10% content of RGD-QS-PEG_3000_-SH show a significantly higher FA area per cell with respect to SAM RGD 100% (65 and 75% increase, respectively) and total FA area per cell medians of 105 and 111 μm^2^, respectively. No significant differences were observed between SAM RGD-QS 1.5 and 10% samples (see Table 2).

Considering that the estimated RGD bulk surface density is at least 2 orders of magnitude lower for the SAM RGD-QS samples than for the SAM RGD 100% (See Table 2), these results indicate that cell adhesion for all substrates featuring hierarchical RGD nanoarchitectonic using quatsomes (SAM RGD-QS) was dramatically higher than for conventional RGD mixed SAMs (SAM RGD). The total FA area per cell increases with the molar ratio of RGD-terminated QSs from 0.125 to 10%, getting values close to FN for 1.5 and 10% (Figures 6 and 7 and Table 2). As more RGD-QS-PEG_3000_-SH are anchored to the SAM, a higher number of cell adhesion peptides are exposed on the surface and higher cell density and FA per cell are observed. However, this trend is not maintained for the SAM RGD-QS 100% surface, where the total FA per cell decreases to a level like that of the SAM RGD 100%. From these results, it seems that the SAM RGD-QS 10% and SAM RGD-QS 1.5% surfaces yield similar cell adhesion, possibly due to an effective saturation of the surface with quatsomes already achieved at a concentration of 1.5%. On the SAM RGD-QS, 0.125% the overall cell adhesion is lower, probably due to a lower effective surface coverage with RGD-QS. It is noteworthy that, according to our estimations, the bulk surface density of RGD in the hybrid SAM RGD-QS 10 and 1.5% is at least more than 1 order of magnitude lower than the bulk surface density of RGD found in the mixed SAMs RGD 100% while yielding higher cell adhesion values.

The positive impact on cell adhesion observed for hierarchical RGD nanoarchitectonic (SAM RGD-QS) on cell adhesion is explained by a few nonexclusive phenomena. The first one is the increase of dimensionality given by the quatsomes and the resulting effective quantity and flexible and fluid disposition of cell adhesion peptides exposed to the cells. Another explanation is that the disposition of cell adhesive ligands in a clustered hierarchical nanostructure instead of a homogeneous distribution enhances their effectiveness for integrin-mediated cell adhesion, as it matches the well-known clustered structure of the FAs.^34^ Substrate rigidity also plays an important role in the growth of focal adhesions,^55^ and quatsomes exhibit Young’s modulus in the order of 10 MPa (see section 5 of the Supporting Information), much lower than that of stiff substrates such as glass, with a Young’s modulus in the range of 50 GPa, which is also beneficial for enhanced cell adhesion. Additionally, a synergistic contribution to the presence of RGD could come from the roughness of the substrate due to the presence of the bulky quatsomes.^12^ However, surfaces functionalized with blank-QSs (Figure 5), which contain the same bulky nanovesicles but do not contain RGD ligands, do not present any significant cell adhesion.

To shed light on these results and further understand some of the observed behaviors like the decrease of adhesion for the SAM RGD-QS 100%, we performed further characterization of the engineered substrates using force spectroscopy with an atomic force microscope (AFM) and electrochemical impedance spectroscopy (EIS).

### AFM Characterization of Hybrid SAMs (RGD-QS)

**Figure 8:**
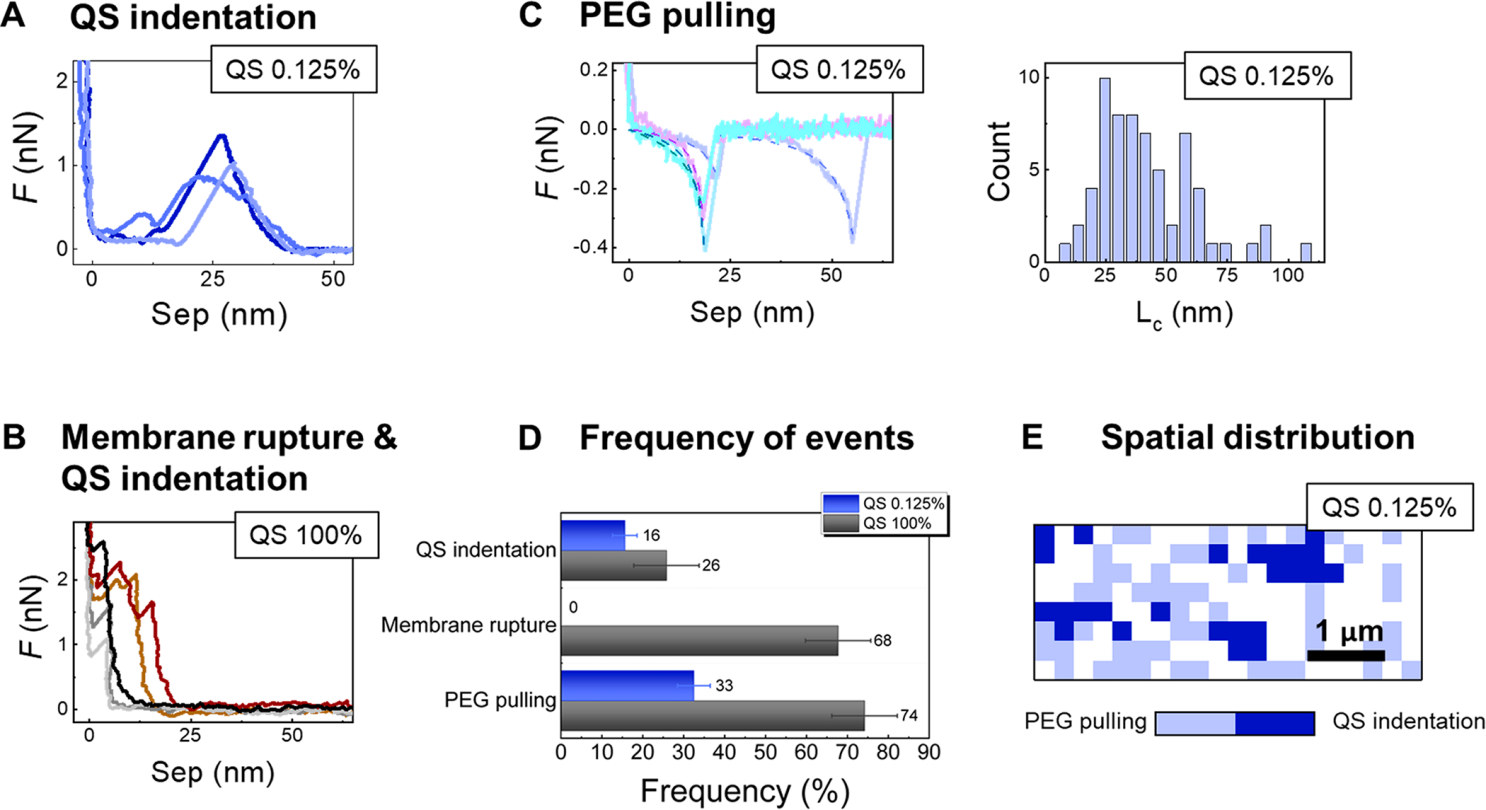
AFM-FS: (A) Representative force vs tip–sample separation approach curves showing QS indentation for SAM RGD-QS 0.125% (with PEG_130_-SH filler). (B) Representative force vs tip–sample separation approach curves showing QS indentation and supported membrane rupture for SAM RGD-QS 100% (without PEG-SH filler). (C) Representative force vs tip–sample separation retraction curves for SAM RGD-QS 0.125% showing PEG_3000_ pulling with WLC fits (left) and the contour length distribution obtained from the WLC fit (right). (D) Fraction of force curves showing QS indentation, supported membrane rupture, and PEG pulling in the SAMs with the presence or absence of PEG-filler molecules. (E) Spatial distribution of positive events QS indentation and PEG pulling, for SAM RGD-QS 0.125%.

Atomic force microscopy-based force spectroscopy (AFM-FS) in liquid was used to obtain insight into the hybrid SAM nanostructures formed by RGD-QSs. The tip–surface approach and retraction force–separation curves were registered in a map mode on different points over a defined surface area. Maps of force–separation curves revealed distinctive features on the SAM RGD-QS 0.125% and SAM RGD-QS 100% substrates (Figure 8D, E): The different kinds of events found on the surfaces were classified as (1) QS indentation and (2) membrane rupture, both observed upon tip–surface approach, and (3) PEG_3000_ pulling (stretching), detected upon retraction. The characteristic force curve profiles are shown in Figure 8A–C. It is important to note that during force map acquisition, the lateral tip movement occurs far away from the surface, contrary to imaging (contact and AC modes) where soft material can be displaced by the scanning AFM tip.

QS indentation events, resulting in cantilever deflection after the AFM tip contacts the nanovesicle while approaching the surface, are found on both SAM RGD-QS 0.125% and SAM RGD-QS 100% substrates. However, the onset of the tip–substrate interaction is different in each case, namely, at a distance of ca. 45 nm from the surface on the hybrid SAM RGD-QS 0.125% (Figure 8A), but at a much shorter distance of maximally ∼25 nm on the SAM RGD-QS 100% (brown and orange curves in Figure 8B). Furthermore, the indentation curves on hybrid SAM RGD-QS 0.125% differ from those of deformed vesicles adhered to a substrate described in the literature.^56^ On the SAM RGD-QS 0.125%, a sudden drop to zero force is observed after vesicle deformation with loading forces up to around 1–1.5 nN, several nanometers before the tip finally enters into contact with the underlying surface. Taken together, these indentation curves suggest that the vesicles in the hybrid RGD-QS 0.125% SAMs mixed with the PEG_130_-SH filler are not adhered to the surface but rather suspended on top, anchored to the Au through the PEG_3000_ linker, allowing them to either break or move aside when a vertical load is applied by the AFM tip.

On the other hand, in SAM RGD-QSs 100% (without PEG-SH filler) the force–separation curves show that the interaction occurs at tip–substrate separations of ∼25 nm (brown and orange curves in Figure 8B). Upon the force increase, at first, a deformation is observed, followed by a rupture event, to finally contact the underlying surface, suggesting that in this case the quatsomes are directly adhered to the surface and highly deformed. Solid supported membrane rupture events can be identified in the force curves through a sudden jump of the tip to the surface at a certain load, and at a distance that corresponds to the membrane thickness of ca. 5 nm (gray and black lines in Figure 8B).^57^ The high occurrence of this event in the absence of PEG_130_-SH filler, i.e., in SAM RGD-QS 100%, (Figure 8B, D) suggests that in direct contact with the gold surface the QSs partly open up and their bilayer spreads out, forming a supported membrane. However, when QSs are mixed with the PEG_130_-SH filler, i.e., in hybrid SAM RGD-QS 0.125%, the PEG_130_-SH filler molecules hinder this direct interaction and prevent the QS from collapsing onto the Au, avoiding the opening and spreading of the QS membrane.

The long PEG chain was further identified, which is a building block of the RGD-QS-PEG_3000_-SH and acts as an anchoring point to the gold substrate. The pulling of this polymer upon tip retraction showed force-dependent extension profiles (stretching) (Figure 8C) that could be fitted by the worm-like chain (WLC) model (dotted lines in Figure 8C).^58^ From the latter, we obtained a distribution of the contour length *L*_C_ (Figure 8C) centered at ∼25 nm, which corresponds to the contour length of SH-terminated PEG_3000_, and a second peak at ∼50 nm, likely resulting from the pulling of two PEG_3000_ molecules (e.g., when indenting on a QSs, one PEG_3000_ anchors the QS to the substrate but there are other PEG_3000_ on the QSs which are not attached to the surface and can be attached to the tip when retracting). These events confirm the robustness of the interaction of the QSs anchoring groups with the gold surface and were found in both SAMs (SAM RGD-QS 0.125% and SAM RGD-QS 100%), with a higher frequency occurring in SAM RGD-QS 100% (Figure 8C, D). The higher frequency of pulling PEG molecules in the SAM RGD-QS 100% can be attributed to the absence of short PEG_130_-SH filler molecules interacting with the surface, thus yielding a higher anchoring of PEG_3000_. Furthermore, the presence of the supported membrane in SAM RGD-QS 100% could increase the amount of PEG_3000_ on the surface due to the exposure of the inner long PEGs from the nanovesicles.

Importantly, in the presence of PEG_130_-SH filler molecules (SAM RGD-QS 0.125%), we observed differentiated patches of “quasi-suspended” QSs (long SH-terminated PEG_3000_ with which the QSs are anchored to the surface) of ≤1 μm^2^ in size (Figure 8E), while in the absence of PEG_130_-SH filler (SAM RGD-QS 100%) practically the whole surface was covered with RGD-containing supported membrane or “adhered” RGD-QSs (Figure 8B,D).

Taking into account all these results, the AFM data suggests that RGD-functionalized QS moieties are neither ruptured nor deformed in the presence of PEG_130_-SH filler, but rather standing up on the SAM surface, in a state of quasi-suspension, likely providing high accessibility to the RGD peptide ligands.

### Electrochemical Characterization of Hybrid SAMs (RGD-QS)

**Figure 9:**
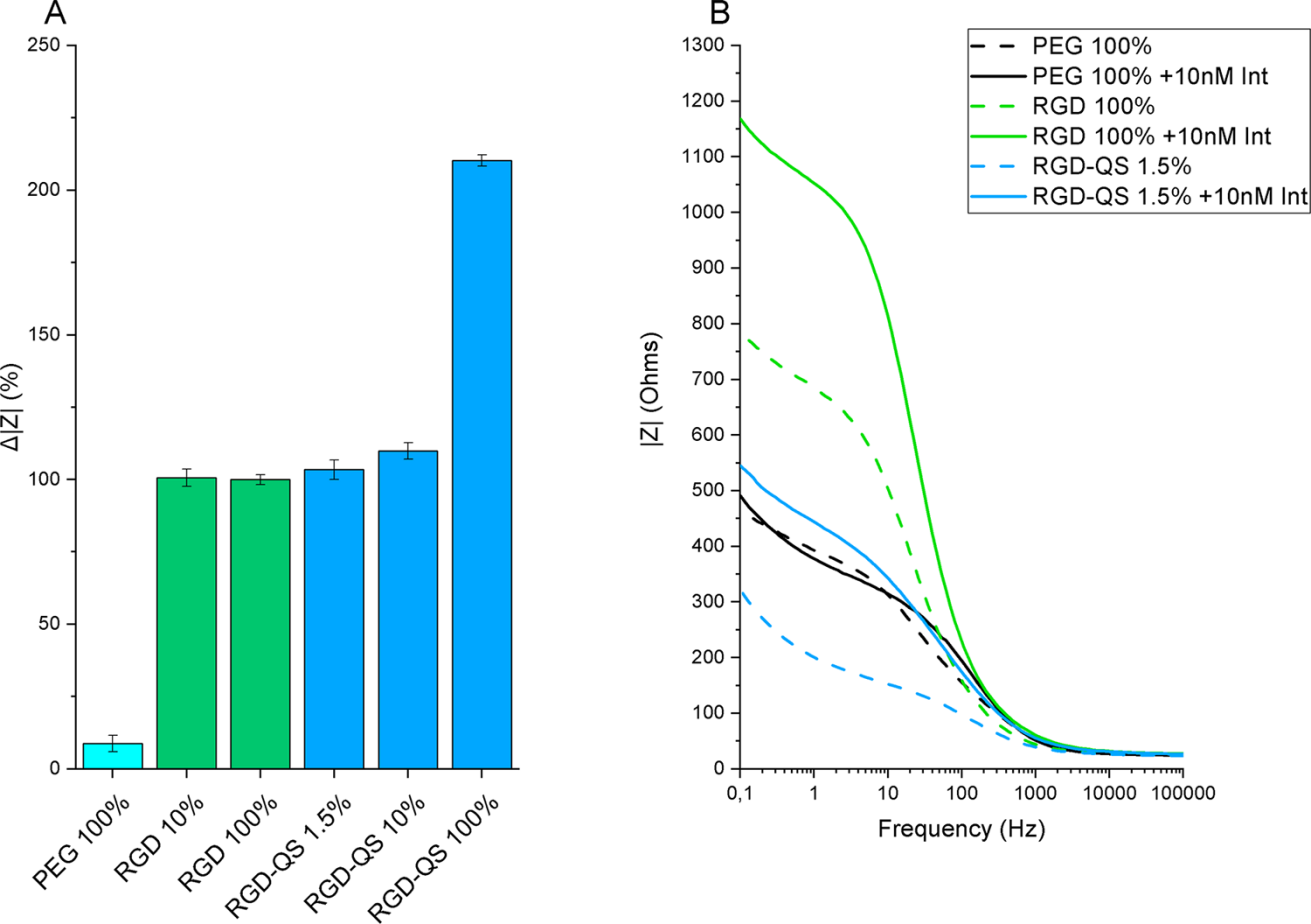
(A) Normalized electrochemical impedance spectroscopy signal (Δ|*Z*| = (|*Z*_*i*_| – |*Z*_0_|)/|*Z*_0_|, where |*Z*_0_| and |*Z*_*i*_| are the impedance modulus before and after integrin interaction, respectively) achieved at each surface for a fixed concentration of integrin (10 nM). (B) Representative Bode magnitude plots (impedance modulus (|*Z*|) vs frequency (*f*)) of three samples before (dashed line) and after incubation with integrin (continuous line). Measurements were done in 0.1 M KCl, 10 mM K_3_[Fe(CN)_6_]/K_4_[Fe(CN)_6_], in a three-electrode configuration using a single junction Ag/AgCl (sat. KCl) as reference electrode, with a Pt wire as the auxiliary electrode and the different SAM surfaces (exposed area: 6 × 10 mm^2^) as working electrodes.

Electrochemical impedance spectroscopy (EIS) is a powerful tool to discriminate changes on the electrode surface derived from small supramolecular interactions by simply monitoring the capability of a redox probe (i.e*.,* [Fe(CN)_6_]^3–/4–^) to be oxidized or reduced on the transducer material throughout a frequency domain.^59^ From an electrochemical point of view, the adsorption of molecules on the electrode surface commonly leads to an insulating layer formation that hinders the interfacial electron transfer kinetics between the redox probe in solution and the electrode, resulting in an impedimetric (|Z|) increase. Accordingly, the electrochemical response of [Fe(CN)_6_]^3-/4-^ on different mixed and hybrid SAM surfaces containing RGD-PEG_260_-SH or RGD-QS-PEG_3000_-SH molecules toward a fixed concentration of integrin in solution was studied. This study enabled us to get information about the accessibility of RGD ligands to interact with the integrin present in the solution. For this aim, the engineered surfaces with different proportions of active RGD sites were investigated (Figure 9).

As expected, the SAM PEG 100% did not present a significant increase in the impedimetric capability after being incubated with integrin when compared to the positive control SAM RGD 100%. Thus, the inability of integrin to interact with the SAM PEG 100% demonstrates that the integrin target must be fully recognized by the RGD ligands via supramolecular RGD-integrin bond formation and that physisorption is not taking place. Importantly, this result reinforces the utility of PEG-SH filler molecules in the composition of mixed and hybrid SAMs. They provide a nonadherent surface in which the specific function of RGD moieties can be studied isolated and serve as a negative control for integrin interaction. Otherwise, the nonsignificant impedimetric differences obtained between the SAM RGD 10% and SAM RGD 100% indicate that the impedimetric response with both surfaces might be saturated and thus, no further change in integrin-surface interaction can be perceived when increasing RGD ligand concentration on the surface of the SAMs from 10 to 100%. It should be noted that the cell adhesion experiments showed that cells were able to yield a different response when cultured in these two substrates (Figures 5 and 7), remarking the ability of cells to take advantage of the difference in RGD density, probably due to integrin clustering in the cell membrane.^60^

Regarding the hybrid SAM surfaces, similar electrochemical behaviors to those on the SAM RGD 100% were observed in both SAM RGD-QS 1.5% and 10%, indicating that the surface response is saturated with a 1.5% RGD-QS-PEG_3000_-SH (see Figure 9). This fact demonstrates excellent accessibility of the RGD exposed on the QS surface. Interestingly, cells behaved similarly in these two conditions in the biological assay, in contrast to the results observed in the 10% and 100% RGD SAMs (see Figure 7). Finally, the highest response obtained by the SAM RGD-QS 100% must be explained due to the different morphology and supramolecular organization of the structured surface. As seen previously in the AFM experiments, on surfaces of SAM RGD-QS 100% the nanovesicles can interact directly with the gold surface, opening the nanovesicles and forming a supported membrane. This fact leads to a closer location of the RGD active molecules on the electrode surface (∼5 nm) in comparison with the RGD-QS anchored with the long PEG tail (25 nm), making it possible to achieve a much more sensitive impedimetric response on the electrode surface. The electrochemical results are in concordance with the ones obtained by AFM since the distance is a key parameter that can alter an impedimetric measurement. Hence, the improved transduction signal achieved at the SAM RGD-QS 100% surface can be explained by the reduced distance between the active sensing object (RGD) and the electrode. All in all, the higher integrin-RGD interactions seen in the RGD-QS samples must be related to higher individual interaction between integrin and its ligand, and not justified by the hierarchical nanostructure of RGD. This is due to the different environments in which integrins are found. In living cells, the hierarchical nanostructuration of RGD appeals to the distribution of integrins on the cell membrane and their natural clustering upon interaction. In this electrochemical experiment, though, integrin is in solution, homogeneously distributed. Thus, the contribution of the clustering of integrin within the cell membrane cannot be evaluated, making the higher signal in the SAM RGD-QS conditions even more remarkable.

## Summary and Conclusions

Suspensions of multifunctional quatsome nanovesicles bearing a long PEG tail with a terminal SH group and RGD ligands were produced to study the impact of the hierarchical nanostructuration of RGD peptides on integrin-mediated cell adhesion. The RGD-QS-PEG_3000_-SH were covalently anchored to gold substrates, forming hybrid SAMs with thiolated PEG_130_-SH fillers. These hybrid SAMs showed good cell adhesion, increasing the total FA area of the adhered cells by ca. 60% in comparison to the SAM containing 100% of RGD-PEG molecules, even though the overall bulk surface density of RGD was at least 2 orders of magnitude lower for the hybrid SAMs containing mixtures of RGD-QS and PEG_130_-SH filler than for the mixed SAM (mixtures of RGD-PEG and PEG_130_-SH). AFM results suggested that the bound nanovesicles in the presence of PEG-SH fillers were in a state of quasi-suspension, anchored to the surface but with some mobility, making the RGD peptide ligands more accessible to the medium and the cells.

An enhancement of cell adhesion was not observed in SAMs made only of RGD-QS-PEG_3000_-SH (SAM RGD-QS 100%), i.e., in the absence of PEG-SH fillers. AFM experiments showed that when 100% of RGD-QS-PEG_3000_-SH were deposited on bare gold the nanovesicles got in contact with the surface and opened up to form supported membranes homogeneously exposing the RGD and thus, losing the hierarchical nanostructure (clustering) of RGD. EIS experiments further confirmed the rupture of the quatsomes upon direct interaction with the bare gold and additionally suggested that integrin may be more prone to interact with RGD when it is anchored in a fluid membrane, as in the case of hybrid RGD-QS SAMs, than on a rigid surface. Due to the free distribution of integrin in solution during the EIS measurements, in comparison with its clustered disposition in the cell membrane to form FAs, no effect of the RGD hierarchic nanostructures using QS was observed electrochemically. These observations corroborate that the hierarchical nanostructuration of RGDs benefits primarily the interaction with integrin in cells.

All these results together point toward the hierarchical nanostructuration of the RGD peptide on surfaces as the cause for the observed strong increase in cell adhesion. Further research in this field could take advantage of the versatility of the robust quatsome platform to study the variation of RGD density within the quatsome surface or the incorporation of a second cell adhesion peptide, allowing an extensive investigation of the cell adhesion mechanisms. This versatility can also be used to exploit different anchoring chemistries to immobilize quatsomes on other materials, and to structure different molecules such as proliferation or differentiation elicitors to better control and tune cell behavior.

Overall, quatsome-based hybrid SAMs opens up many possible pathways for the understanding of cell behavior, which is not limited just to 2D surfaces but could also be applied to 3D scaffolds, improving the performance of clinical applications like implants and tissue engineering.

## Experimental Section

### RGD-QS-PEG-SH Preparation

The equipment used to produce the nanovesicle formulations through the DELOS-susp method is a small-scale reactor that has been previously described.^43^ Chol-PEG_200_-c(RGDfK) (RGD-PEG) was prepared as described previously:^43,53^ 9.28 μmol were dissolved in 800 μL of DMSO before adding 2.08 mL of an ethanolic solution dropwise with 69.6 μmol of cholesterol (Panreac, Barcelona, Spain) and 0.27 μmol of chol-PEG_3000_-SH (Nanocs, New York, USA). The resulting mixture of the three cholesterol derivatives with a molar ratio of chol/chol-PEG_200_-c(RGDfK)/chol-PEG_3000_-SH of 260/30/1 was loaded into a 7.5 mL high-pressure vessel and volumetrically expanded with compressed CO_2_ to reach a working pressure of 11.5 MPa. Then, the system was kept at 308 K and 11.5 MPa for approximately 1 h to achieve complete homogenization and thermal equilibration. Later on, 157.7 μmol of myristalkonium chloride (MKC; US Biological, Salem, United States) were added to 24 mL of ultrapure H_2_O (Milli-Q Advantage A10 water purification system, Millipore Ibérica, Madrid, Spain) and the expanded organic phase was depressurized over the aqueous solution at RT. In this step, a flow of N_2_ at the working pressure was used as a plunger to push down the CO_2_-expanded solution from the vessel and to maintain a constant pressure of 11.5 MPa inside the vessel during depressurization. The resulting suspensions of nanovesicles were stored at 4 °C until their characterization. The remains of organic solvent and excess MKC of such suspensions were removed and substituted by ultrapure H_2_O using the KrossFlo Research IIi TFF diafiltration system (KR2i) (Spectrum Laboratories, SL) following the procedure already described in previous work.^47^ Briefly, a 100 kDa cutoff mPES hollow fiber (Repligen, USA) column was used. Ten milliliters of the suspension was submitted to six cycles of diafiltration with ultrapure water (60 mL), resulting in the elimination of the remaining organic solvents present in the sample. See Section 1 of the Supporting Information for the formulas of the employed molecules.

### Quantification of Cholesterol and Cholesterol-PEG_200_-c(RGDfK) in RGD-QS-PEG_3000_-SH Formulations

For the quantitative analysis of cholesterol and the chol-PEG_200_-c(RGDfK) molecules in the RGD-QS-PEG-SH, an HPLC (1100 series, Agilent Technologies, USA) coupled to an Evaporative Light Scattering Detector (ELSD, 1260 infinity ELSD, Agilent Technologies, USA) was employed. Before sample injection 1 mL of quatsome formulation was lyophilized and dissolved in 1 mL of methanol to obtain a suitable solution for chromatographic analysis. Cholesterol and chol-PEG_200_-c(RGDfK) separation were carried out using a C18 Symmetry (5 μm; 4.6 × 150 mm) column (Waters Cromatografía S.A., Spain) with an ELSD nebulization temperature of 40 °C and evaporative temperature of 80 °C. The mobile phase was a mixture of methanol with water (95:5) (phase mobile A, MPA) and formic acid in isopropanol (0.1% HCOOH) (mobile phase B, MPB) using elution conditions described in Table 3. Two microliters of freeze-dried quatsomes were injected in the HPLC-ELSD. The analysis was carried out in triplicate for each quantification.

**Table 3:** Gradient Elution Method for the Quantitative Analysis of Cholesterol and Chol-PEG_200_-c(RGDfK)

time (min)	MPA (%)	MPB (%)	flow(mL/min)
3	97	3	1
4	88	12	2
16	88	12	2
16.5	95	5	2
19	97	3	2
19.5	97	3	1

### Size Distribution and Surface Charge of Quatsomes

Size and polydispersity index (PdI) of multifunctional quatsomes were determined using the dynamic light scattering (DLS) technique, while the apparent ζ-potentials using the electrophoretic light scattering (ELS) technique, applying the Helmholtz–Smoluchowski equation. Both measurements were carried out using Zetasizer Nano ZS equipment, which has a noninvasive backscatter technology (NIBS) (Malvern Panalytical, UK). The measurements were done at 25 °C, using 1 mL of the samples without previous treatment or any dilution, and a solvent correction was applied depending on the volume fraction of ethanol in the dispersant. The reported values are the average of three consecutive measurements on the same sample using the Zetasizer Software. Size and PdI data were based on the intensity size distribution and correspond to the *z*-average (± standard deviation) between the three measurements.

### Cryo-Transmission Electron Microscopy of Multifunctional Quatsomes

The size, morphology, and homogeneity of the multifunctional quatsomes were studied using cryogenic transmission electron microscopy (Cryo-TEM). Samples were vitrified in a controlled environment system (EMCPC, Leica Microsystems, Germany). A 2–4 μL drop of the sample was placed in a copper grid coated with a perforated polymer film. After 30 s, the sample excess was removed by blotting (1–2 s) with filter paper to obtain a thin film of 20–400 nm. Immediately after, the grid was plunged into liquid ethane at 94 K. The vitrified sample was kept cool (77 K) during the transfer procedure to the microscope, as well as during the image acquisition, which was done with a JEOL JEM-2011 microscope (JEOL LTD, Tokyo, Japan) operating at 120 kV.

### Mixed and Hybrid SAM Preparation on Gold Surfaces

Substrates for cell culture experiments were produced as follows. Thin glass coverslips were cleaned in piranha solution [concentrated H_2_SO_4_, aqueous H_2_O_2_, 3:1] for 45 min, rinsed with ultrapure water, and dried under a nitrogen stream. A 3 nm adhesion layer of titanium and a subsequent 8 nm layer of gold were deposited on the glass substrates using vapor deposition equipment (Edwards Auto 306; Edwards, Crawley, UK). Substrates for electrochemical impedance spectroscopy (EIS) consist of gold-covered glass substrates of 1.1 mm thickness coated with a 200 nm layer of gold (Ssens, Enschede, The Netherlands). Substrates for AFM measurements consist of template stripped flat gold. For this, 200 nm of Au were evaporated on freshly cleaved mica without any adhesion layer. Afterward, thoroughly cleaned, piranha etched glass support was glued to the Au, using epoxy glue (EPO-TEK 353ND, Epoxy Technology, USA). The epoxy glue was cured at 150 °C for 1–2 h. Immediately before the experiment, the flat Au surface was exposed by mechanically stripping off the mica.

Before SAM deposition, the different gold-covered glass substrates were consecutively cleaned through 5 min sonication in HPLC grade dichloromethane, acetone, and ethanol. Coverslips were afterward cleaned in a UV ozone cleaner (UVO-cleaner: model 42 series; Jelight company, USA) for 20 min. Then, the coverslips were incubated with aqueous solutions of the desired components to form either the mixed SAMs or the hybrid SAMs. For mixed SAMs preparation, an RGD-terminated PEG molecule [Cys_3_-EG_6_-c(RGDfE)], being EG = −CH_2_–CH_2_–O–, (RGD-PEG_260_-SH) (PSL Peptides, Heidelberg, Germany) was combined with the PEG-filler molecule [HS-(CH_2_)_8_-EG_3_-OH] (Prochimia, Sopot, Poland) (PEG_130_-SH) in four different molar ratios of PEG_130_-SH:RGD-PEG_260_-SH 0:100, 1:99, 10:90, and 100:0, namely SAM RGD *x*% (*x* = 0, 1, 10, and 100). On the other hand, for hybrid SAMs preparation, RGD-functionalized thiolated quatsomes (RGD-QS-PEG_3000_-SH) were combined with the PEG-filler molecule in four different molar ratios of PEG_130_-SH:RGD-QS-PEG_3000_-SH 6600:1, 6000:1, 660:1, and 0:1, namely, SAM RGD-QS *x*% (*x* = 0.125, 1.5, 10, and 100). For RGD-QS hybrid SAMs, the molar ratios with PEG were calculated by assuming that RGD-QS-PEG_3000_-SH were individual entities. Calculations of RGD-QS concentration and their surface RGD density can be found in Section 3 of the Supporting Information.

### Cell Culture, Seeding, and Immunostaining

Human osteosarcoma cells (U2OS) were obtained from the American Type Culture Collection (ATCC; Manassas, VA, USA). Cells were routinely cultured in Dulbecco’s Modified Eagle’s medium (DMEM; Thermo Fisher Scientific, USA) supplemented with 10% fetal bovine serum (FBS) and 1% penicillin/streptomycin in a humidified atmosphere containing 10% CO_2_ at 37 °C.

Cells were grown on mixed and hybrid SAMs. Specifically, U2OS cells were seeded at a concentration of 22500 cells/cm^2^ in a DMEM medium with 10% FBS. Plates were incubated at 37 °C and 10% CO_2_ for 24 h.

Thereafter, cells were fixed by the addition of 4% formaldehyde for 20 min. After fixation, cells were permeabilized by adding 0.1% Triton in PBS and treated with a blocking solution (1% bovine serum albumin in PBS) for 30 min to prevent nonspecific binding. After blocking, substrates were incubated for 1 h at RT with a mouse monoclonal antipaxillin antibody (Sigma-Aldrich, USA) diluted at 1:400. After incubation with the primary antibody, samples were washed with PBS for 10 min, then they were incubated 45 min at RT with the secondary antibody Alexa Fluor 488 goat antimouse IgG (1:100; Thermo Fisher Scientific, USA) and with the Hoechst dye (1:1000; Thermo Fisher Scientific, USA). Primary and secondary antibodies were diluted in the blocking solution. Finally, samples were washed with PBS for 10 min and mounted with ProLong Gold Antifading Mountant (Thermo Fisher Scientific, USA).

### Confocal Microscopy

Images for the cell adhesion analysis were acquired with a Leica TCS SP5 AOBS spectral confocal microscope (Leica Microsystems, Mannheim, Germany) with an HCX PL APO CS 20× objective and an HCX PL APO lambda blue 63× objective. Nuclei and FA staining were excited with a diode UV laser beam at 405 nm and an argon laser beam at 488 nm, respectively, and detected at 758–800 and 717–758 nm, respectively. Images for cell adhesion analysis were acquired from three independent experiments.

### Image Analysis

FA quantification and cell density data were extracted from microscopy images, using the *ImageJ* software (National Institute of Health, USA). Data were treated in *Origin* (OriginLab, USA) and MS Excel.

### Cell Density

Images of the nuclei were pretreated to increase the definition of the structures. Afterward, images were turned into binary with the threshold tool, and the “Analyze Particles” function was used to measure the cell count in each image. The microscopy field dimensions were taken into account to calculate the cell density for each image.

### Focal Adhesion Total Area

Images were pretreated with contrast enhancement and a mean filter to increase the structure definition. Afterward, images were turned into binary with the threshold tool, and the “Analyze Particles” function was used to measure the number and area of the FAs present in the cells.

### Atomic Force Microscopy and Spectroscopy of Hybrid SAMs

Atomic force microscopy-based force spectroscopy (AFM-FS) experiments were performed using an MFP-3D atomic force microscope (Asylum Research, Oxford Instruments) and V-shaped Si_3_N_4_ cantilevers with Si tips and nominal spring constants of 0.1 N/m (SNL, Bruker AFM Probes). AFM images over areas from 0.5 × 0.5 to 5 × 5 μm^2^ were acquired in contact and AC mode but no topographical features were obtained, probably because of sample deformation or rupture. AFM-FS was performed by approaching and retracting the AFM tip to the sample at a constant velocity of 1 μm/s. Maps of force–separation curves were recorded over areas of 5 × 5 μm^2^ by following an array of points of 20 × 20 (force map mode). All experiments were performed at RT and in a liquid environment (Milli-Q water).

### Electrochemical Impedance Spectroscopy

Electrochemical impedance spectroscopy (EIS) was performed on a Novocontrol Alpha-AN impedance analyzer with a potentiostat POT/GAL 30 V/2A electrochemical interface using a conventional three-electrode configuration cell filled with 20 mL of a 0.1 M KCl solution containing 10 mM K_3_[Fe(CN)_6_]/K_4_[Fe(CN)_6_] as a redox marker. The electrode configuration was a single junction Ag/AgCl (sat. KCl), as a reference electrode, a Pt wire, as an auxiliary electrode, and the different SAM surfaces (exposed area: 6 × 10 mm^2^), as working electrodes. Impedimetric experiments were obtained using the following conditions: frequency range from 100 kHz to 100 mHz; bias potential of +150 mV and AC amplitude of 5 mV. All the experiments were performed at room temperature and under environmental conditions. EIS data was represented as a Bode magnitude plot (impedance modulus (|*Z*|) vs frequency (*f*)). Measurements were carried out per triplicate with three different electrodes (*n* = 9) to study both repeatability and reproducibility. Surfaces were measured before and after incubation with integrin 10 nM (Recombinant human integrin αV β3, Bio-Techne, USA). The normalized signal for each sample was obtained from the change of the impedance modulus (|*Z*|) at 100 mHz after incubation with integrin.

## Supporting Information Available

Multifunctional quatsome nanovesicle (RGD-QS-PEG3000-SH) production, self-assembly monolayer (SAM) production, calculations for the estimation of *M*_W_ and molar concentration of quatsomes, calculations for average surface RGD density and average RGD to RGD distance of mixed and hybrid RGD-presenting SAMs, and Young’s modulus of quatsomes in quasi-suspension (PDF)

## Abbreviations

RGD: cyclic peptide c(RGDfK) that contains Arg-Gly-Asp
PEG: poly(ethylene glycol)
SAM: self-assembled monolayer
FA: focal adhesion
QS: quatsome
