# Supplementary material for: Hierarchical Quatsome-RGD Nanoarchitectonic Surfaces for Enhanced Integrin-Mediated Cell Adhesion
Source: ACS Appl Mater Interfaces. 2022 Oct 17;14(42):48179–93. doi: 10.1021/acsami.2c10497 (PMC9614722; doi:10.1021/acsami.2c10497)
Supplement: Supplementary file 1 — am2c10497_si_001.pdf [file am2c10497_si_001.pdf]

Supporting information

Hierarchical Quatsome-RGD nanoarchitectonic surfaces for enhanced integrin-mediated cell adhesion

Marc Martínez-Miguel[1,2]; Miquel Castellote-Borrell[1]; Mariana Köber[1,2]; Adriana R. Kyvik[1,2]; Judit Tomsen-Melero[1,2]; Guillem Vargas-Nadal[1]; Jose Muñoz[1]; Daniel Pulido[2,3]; Edgar Cristóbal-Lecina[2,3]; Solène Passemard[1]; Miriam Royo[2,3]; Marta Mas-Torrent[1,2]; Jaume Veciana[1,2]; Marina I. Giannotti[2,4,5]; Judith Guasch[1,2,6]; Nora Ventosa[1,2,*]; Imma Ratera[1,2,*]

[1] Institut de Ciència de Materials de Barcelona (ICMAB-CSIC), Campus UAB, 08193, Bellaterra, Spain

[2] Biomedical Research Networking Center on Bioengineering, Biomaterials and Nanomedicine (CIBER-BBN), Spain

[3] Institut de Química Avançada de Catalunya (IQAC-CSIC), 08034, Barcelona, Spain

[4] Nanoprobes and Nanoswitches group, Institute for Bioengineering of Catalonia (IBEC), The Barcelona Institute of Science and Technology (BIST), 08028, Barcelona, Spain

[5] Departament de Ciència dels Materials i Química Física, Universitat de Barcelona, 08028, Barcelona, Spain

[6] Dynamic Biomimetics for Cancer Immunotherapy, Max Planck Partner Group, ICMAB-CSIC, Campus UAB, 08193, Bellaterra, Spain

Corresponding Authors:

*Imma Ratera. iratera@icmab.es *Nora Ventosa. ventosa@icmab.es

Contents

1. Multifunctional Quatsome nanovesicle (RGD-QS-PEG_{3000}-SH) production
2. Self-Assembly Monolayers (SAMs) production
3. Calculations for the estimation of M_W and molar concentration of Quatsomes
4. Calculations for average surface RGD density and average RGD to RGD distance of mixed and hybrid RGD-presenting SAMs
5. Young's modulus of Quatsomes in quasi-suspension
6. References

1. Multifunctional Quatsome nanovesicle (RGD-QS-PEG$_{3000}$-SH) production

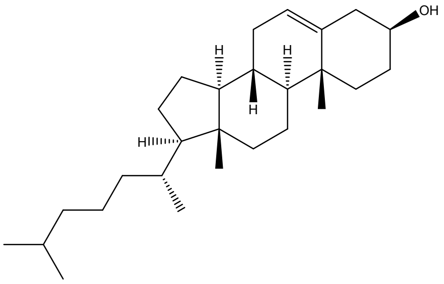

A

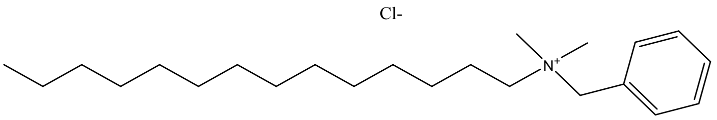

B

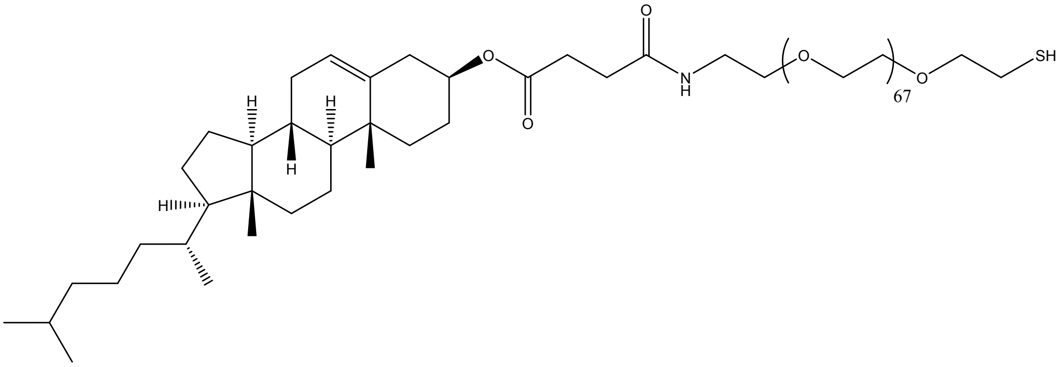

C

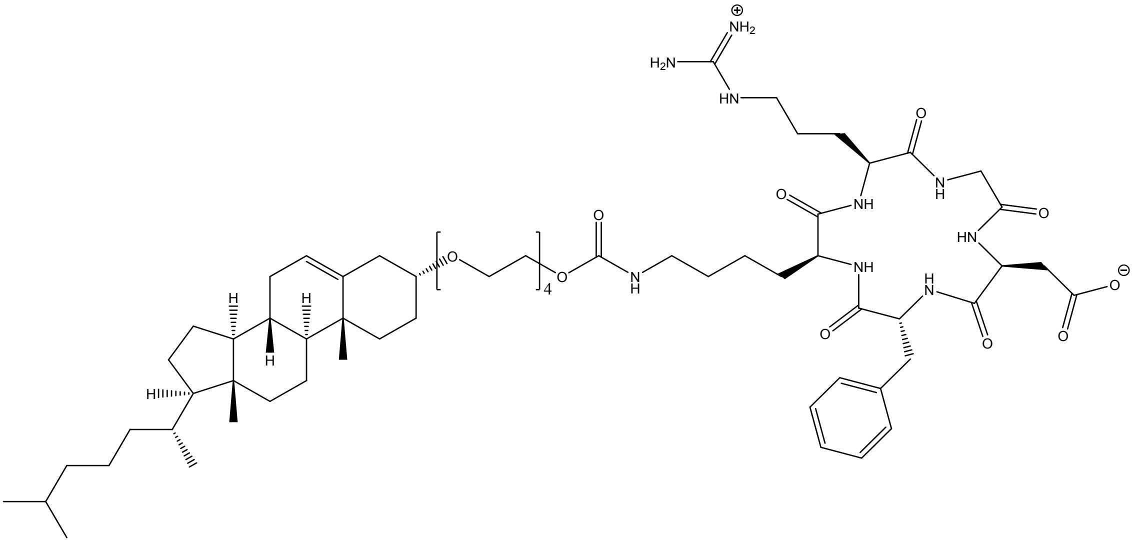

D

Figure S1. Molecules used for the production of RGD-decorated nanovesicles (RGD-QS-PEG$_{3000}$-SH). **A.** Cholesterol. **B.** Myristalkonium chloride (MKC). **C.** Chol-PEG$_{3000}$-SH **D.** Chol-PEG$_{200}$-c(RGDfK).

A

B

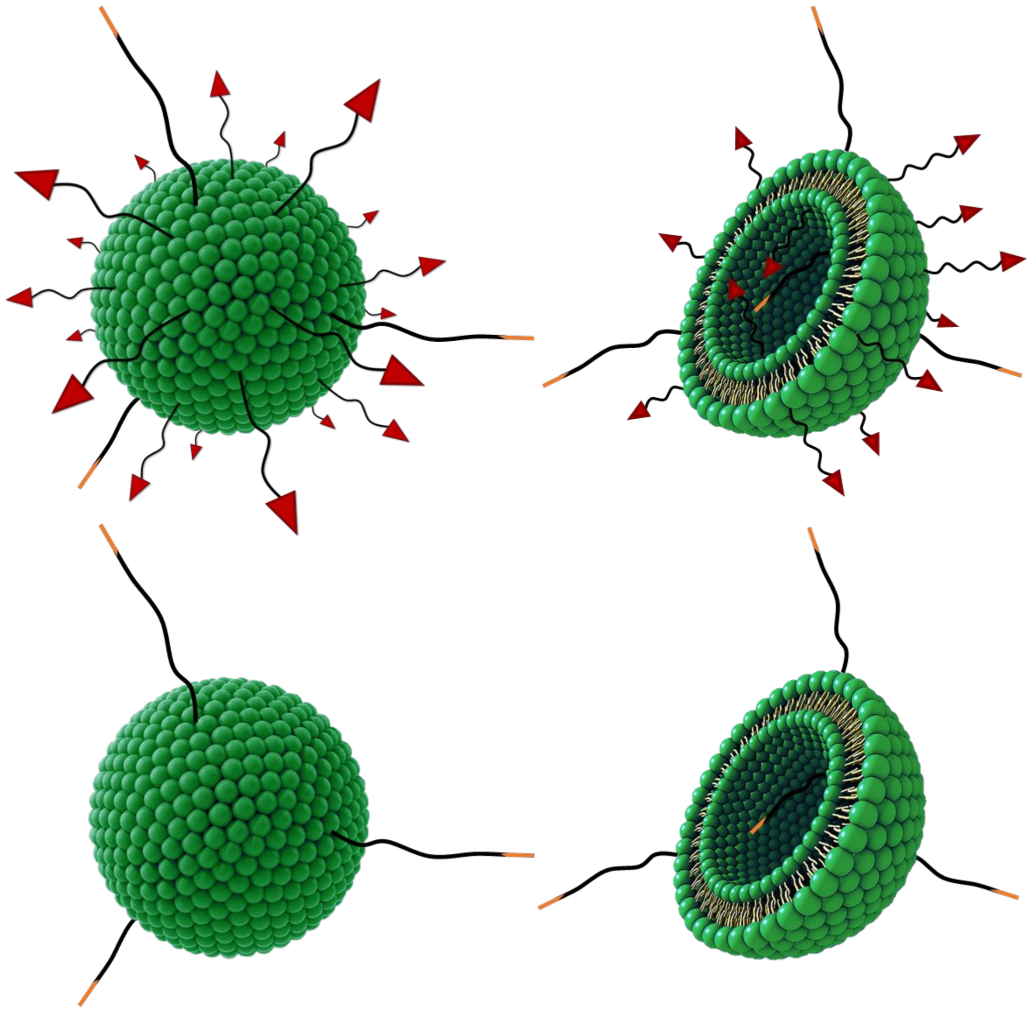

Figure S2. Schematic representation of the produced Quatsome nanovesicles composed of **A.** cholesterol, MKC, chol-PEG$_{200}$-c(RGDfK) (black chain with red triangle) and chol-PEG$_{3000}$-SH (black chain with orange end) and of **B.** cholesterol, MKC and chol-PEG$_{3000}$-SH (black chain with orange end). Notice that, as seen in the cross-sections, some of the functionalized molecules can be oriented into the inner space of the Quatsome.

2. SAM production

A

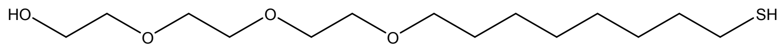

B

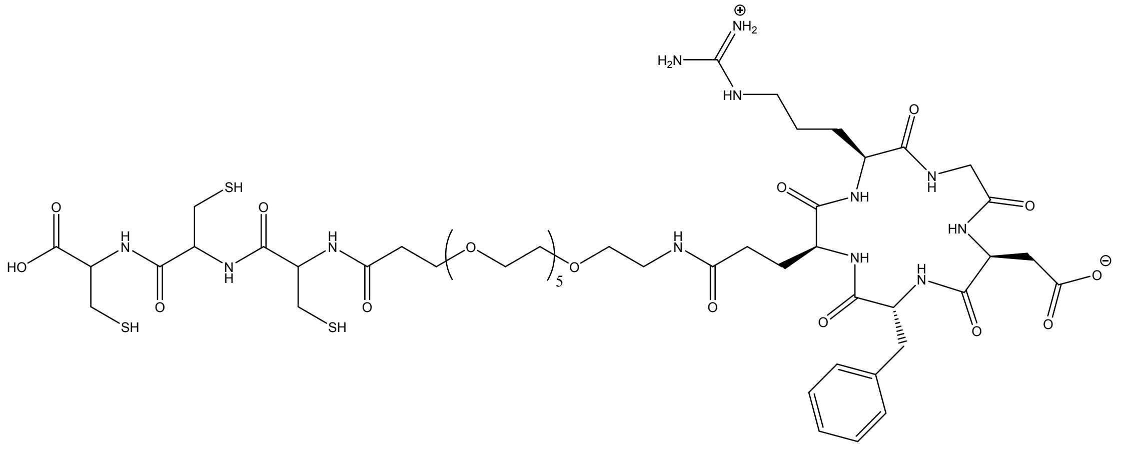

Figure S3. Molecules used for the production of mixed SAMs and the production of hybrid SAMs. **A.** HS-$(CH_2)_8$-EG_3-OH (PEG_{130}-SH filler). **B.** Cys_3-EG_6-c(RGDfE) (RGD-PEG_{260}-SH).

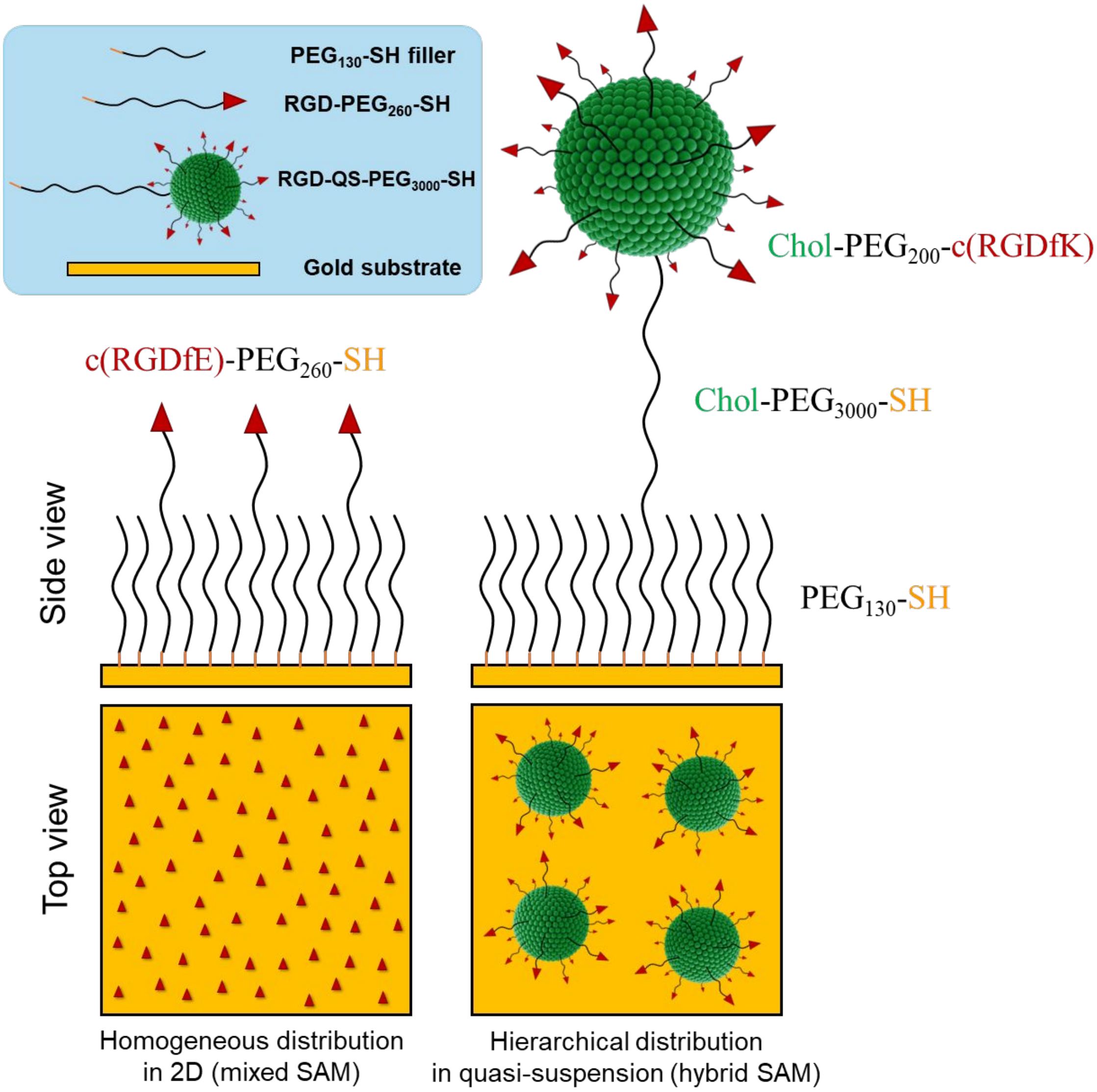

Figure S4. Schematic representation of the two kinds of substrates produced. On the left, a mixed SAM featuring thiolated PEG_{130}-SH (PEG-filler) and thiolated RGD-terminated PEG (RGD-PEG_{260}-SH). On the right, a hybrid SAM composed of thiolated PEG_{130}-SH (PEG-filler) and thiol-functionalized RGD-quatsomes (RGD-QS-PEG3000-SH). Both SAMs are depicted with the same bulk average surface RGD density.

3. Calculations for the estimation of M_W and molar concentration of Quatsomes

The average Quatsome M_W in the produced formulation was estimated by dividing the mass of nanovesicle membrane components by the number of nanovesicles in 1 mL of nanovesicle formulation.

1) The mass of nanovesicle membrane components in 1 mL was measured by triplicate by weighting the lyophilized product of 1 mL of nanovesicle formulation prepared as detailed in the experimental section of the main text. For the lyophilization process a lyophilizer (LyoQuest, Telstar) was used. Samples were lyophilized at -80 °C and 0.03 mBar for 96 h.
2) The particle concentration on the nanovesicular formulation was measured using Multi-angle Dynamic Light Scaterring (MADLS) (Zetasizer Ultra, Malvern Panalytical).

The formula used to estimate the average mass per quatsome nanovesicle was the following one:

$$QS\ M_W = \frac{concentration\ of\ membrane\ components\ \left(\frac{mg}{mL}\right)}{concentration\ \ of\ particles\ \left(\frac{no.particles}{mL}\right)} \#(1)$$

The resulting M_W for QS was $1.86 \cdot 10^7 \pm 0.78 \cdot 10^7$ Da, which allowed us to estimate the molar concentration of the QS formulations from a known mass and volume of sample to 0.38 nM.

4. Calculations for average surface RGD density of mixed and hybrid RGD-presenting SAMs

“Bulk” average surface RGD density is defined as average number of RGD units per area, considering a homogeneous distribution of the RGD units over the area.

“Local” average surface RGD density is determined by the average RGD to RGD distance.

I) Bulk and local average surface RGD density and average RGD to RGD distance in mixed SAMs

Due to the nature of the mixed SAM, in which RGD peptides are homogeneously distributed along the 2D surface, both bulk and local average surface RGD densities are equal independently of dilution (see **Figure 4** in the main text).

For the calculation of RGD density in mixed SAMs, the bulkiness of the cyclic RGD peptide was considered. The whole HS-PEG_{260}-RGD molecule used to functionalize gold surfaces was modeled on the PyMOL software (The PyMOL Molecular Graphics System, Version 2.0 Schrödinger, LLC.) and its projected circular area on a surface was estimated to be 0.74 nm^2.

On a surface saturated with RGD (see **Figure S5**), the RGD density would be:

$$RGD\ density\ (SAM\ RGD\ 100\%\ ) = \frac{1\ \mathrm{RGD\ unit}}{RGD\ area}\cdot\eta = \frac{1\ \mathrm{RGD\ unit}}{0.74\ nm^2}\cdot 0.9 = 1.22\frac{RGD}{nm^2}\#(2)$$

Due to the approximation of the RGD units to circular areas, a factor of 0.9 was applied as the maximum packing density “η” of circles in a hexagonal lattice,[1] since a packing of circles is bound to leave some void space between units.

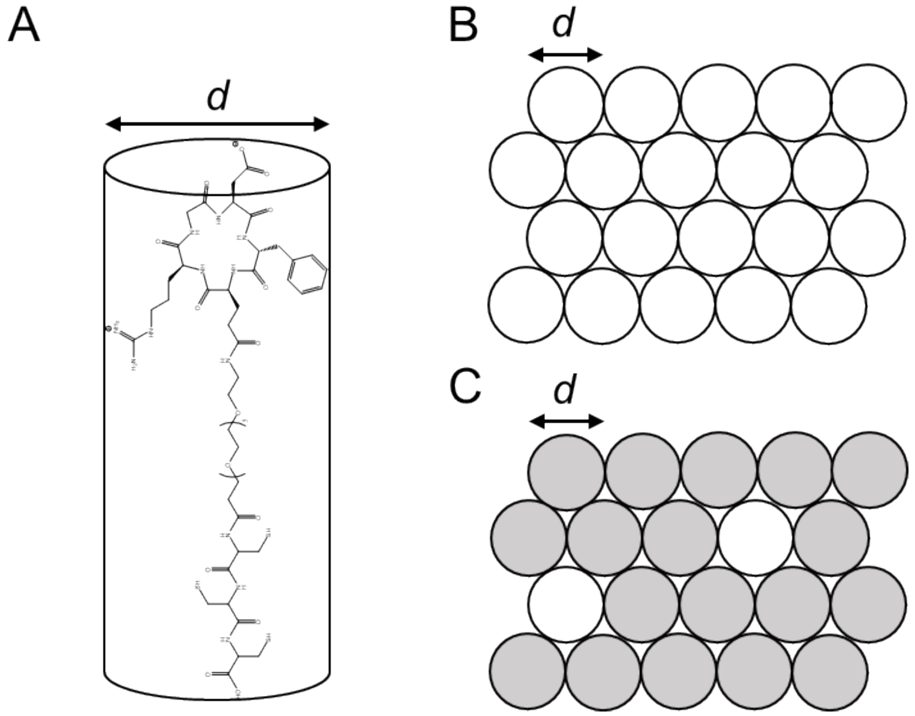

Figure S5. A. Representation of the projected circular area of the RGD-PEG_{260}-SH molecule with a certain diameter "*d*". **B.** Top-down schematic representation of the arrangement of RGD-PEG_{260}-SH molecules as a densely packed circular pattern with a certain diameter "*d*". **C.** Top-down schematic representation of the dilution of RGD-PEG_{260}-SH molecules (white circles) in a 1:9 ratio with non-RGD presenting units (grey circles).

Mixed SAMs feature decreasing molar ratios of RGD versus a PEG_{130}-SH filler molecule. In those diluted samples, average surface RGD densities were estimated to decrease according to the molar ratios (see **Figure S5 C**).

$$RGD\ density\ (mixed\ SAM\ RGD\ 10\%\ ) = 1.22\,\frac{RGD}{nm^2}\cdot 0.1 = 0.122\frac{RGD}{nm^2}\#(3)$$

$$RGD\ density\ (mixed\ SAM\ \ RGD\ 1\%) = 1.22\,\frac{RGD}{nm^2}\cdot 0.01 = 0.0122\frac{RGD}{nm^2}\#(4)$$

Considering the densities estimated in the previous section, an Area of Influence (AoI) can be calculated from each density to find the average circular area in which an RGD molecule is found at the center.

$$Area\ of\ Influence\ (nm^2) = \frac{1}{RGD\ surface\ density\ \left(\frac{RGD\ units}{nm^2}\right)} \#(5)$$

Thus, the average distance (*d*) between RGD units at the center of these circular areas, when arranged on a surface, is double the radius of the estimated AoI.

$$r = \sqrt{\frac{Area}{\pi}} \#(6)$$

$$d = 2\cdot\sqrt{\frac{Area\ of\ Influence}{\pi}} = 2\cdot\sqrt{\frac{1}{RGD\ surface\ density\cdot\ \pi}} \#(7)$$

Considering the estimated RGD densities of each mixed SAM, the resulting average RGD-RGD distances are calculated as follows:

$$d(RGD100\%) = 2\cdot\sqrt{\frac{1}{1.22\pi}} = 1.02\ nm\ \#(8)$$

$$d(RGD10\%) = 2\cdot\sqrt{\frac{1}{0.12\pi}} = 3.25\ nm\ \#(9)$$

$$d(RGD1\%) = 2\cdot\sqrt{\frac{1}{0.01\pi}} = 11.28\ nm\ \#(10)$$

II) Bulk and local average surface RGD density and average RGD to RGD distance in QS-based hybrid SAMs

When dealing with hybrid SAMs, the bulk and local average surface RGD densities behave differently when the sample is diluted. Due to dilution, the bulk average surface RGD density will decrease as the overall amount of RGD per area is reduced. However, the average RGD to RGD distance will not vary due to the RGD peptides being retained

on the QS membrane. Thus, even with dilution, the local average surface RGD density remains constant (see **Figure 4** in the main text).

To calculate the local average surface RGD density, the density of RGD on the membrane of the RGD-QS-PEG_{3000}-SH nanovesicle was considered. The Quatsome membrane is composed by sterol-surfactant bimolecular synthons[2,3] with an estimated polar head area of 0.58 nm^2, according to molecular dynamics simulations.[2] In this work, the surfactant is myristalkonium chloride (MKC), which forms a synthon with a polar head area of the same magnitude as the one previously reported for cholesterol-CTAB synthons.

From the HPLC-ELSD measurements the final concentrations of cholesterol and cholesterol-PEG200-c(RGDfK) in the RGD-QS-PEG3000-SH nanovesicle formulations were 1.930 ± 0.045 mM and 0.106 ± 0.001 mM respectively. These measurements reveal that the content of cholesterol-PEG_{200}-c(RGDfK) is 5% molar of the total cholesterol in the nanovesicular formulation.

Thus, the RGD density within the Quatsome membrane can be calculated as the product of the synthon surface density (synthon unit / synthon area) by the proportion of synthons that carry an RGD peptide due to the MKC being coupled to a cholesterol-PEG_{200}-c(RGDfK) instead of a regular cholesterol (see **Figure S6**). Due to the approximation of the RGD units to circular areas, a factor of 0.9 was applied as the maximum packing density "η" of circles in a hexagonal lattice.[1]

$$\boldsymbol{QS\ membrane\ RGD\ density} = \frac{1\ \text{synthon unit}}{synthon\ area} \cdot \eta \cdot \%\ chol_{cRGDfk} = \frac{1\ \text{synthon unit}}{0.58\ nm^2} \cdot 0.9 \cdot 0.05 = 0.078 \frac{RGD}{nm^2} \#(11)$$

The estimated local density would also be the bulk average surface RGD density in a substrate saturated with Quatsomes. Thus, the bulk average surface RGD density in these substrates cannot be higher than the local density of RGD in the membrane.

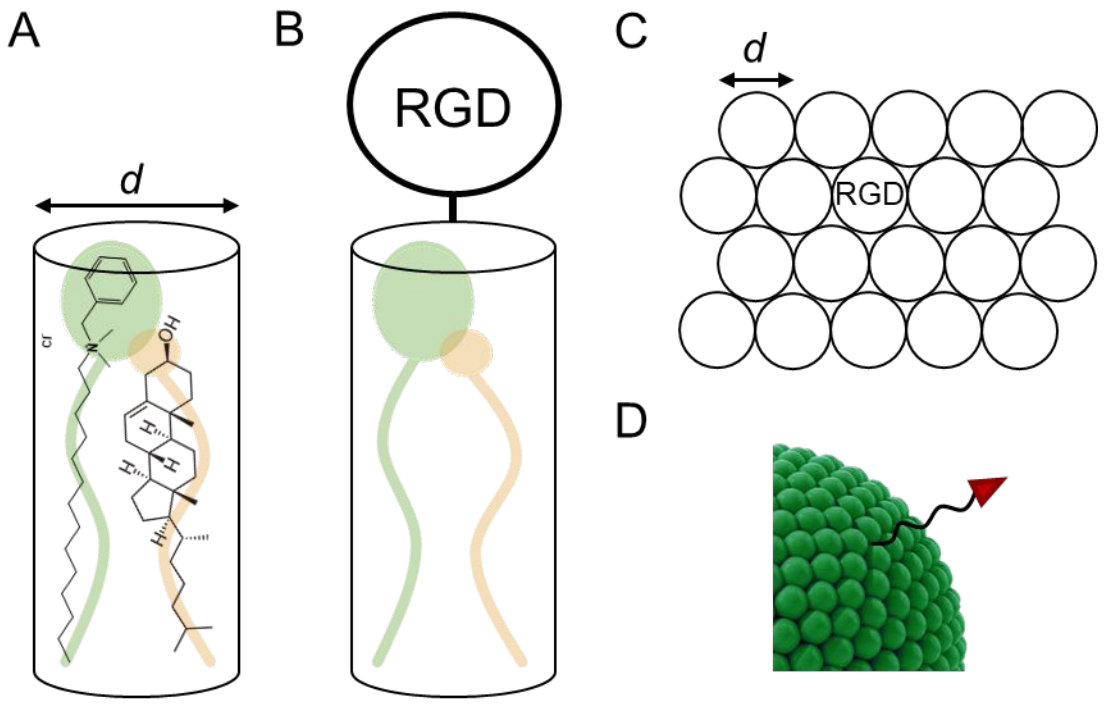

Figure SI6. A. Schematic representation of the synthon, composed of cholesterol and MKC, and its polar head, which features a circular area with a certain diameter "*d*". **B.** Schematic representation of a synthon with an RGD peptide. **C.** Top-down schematic representation of the Quatsome membrane as an arrangement of the circular synthon areas with a certain diameter "*d*" in which 1 every 20 synthons features an RGD peptide. **D.** Representation of the previously represented schematic theoretical area on the membrane of a Quatsome.

The QS-based hybrid SAMs were produced by incubating mixtures of RGD-QS-PEG_{3000}-SH and PEG_{130}-SH in various molar ratios. In those diluted samples, bulk average surface RGD densities can be estimated to decrease according to the molar ratios.

$$RGD\ density\ (RGD - QS\ 10\%) = 0.078\frac{RGD}{nm^2}\cdot 0.1 = 0.0078\frac{RGD}{nm^2} \#(12)$$

$$RGD\ density\ (RGD - QS\ 1.5\%) = 0.078\frac{RGD}{nm^2}\cdot 0.015 = 0.0012\frac{RGD}{nm^2}\#(13)$$

$$RGD\ density\ (RGD - QS\ 0.125\%) = 0.078\frac{\mathrm{RGD}}{nm^2}\cdot 0.00125 = 0.0001\frac{RGD}{nm^2}\#(14)$$

However, the previously provided values of RGD density are very low and could underestimate the real presence of RGD on the sample. To approach this RGD density from another angle, an estimation of the average Quatsome to Quatsome distance in these SAMs was also made by using the same approach as in the RGD to RGD distance. Quatsomes were assumed to bind to the surface through long -SH terminated molecules packed with a density similar to that of the RGD molecules (1.22 molecules/nm^2).

$$Quatsome\ to\ Quatsome\ distance = 2\sqrt{\frac{1}{\pi\cdot Surface\ Density}}\ \#(15)$$

$$10\%\ Quatsome\ SAM \rightarrow d =\ 2\sqrt{\frac{1}{\pi\cdot\left(1.22\,\frac{molecules}{nm^2}\cdot 0.1\right)}} = 3.23\ nm\ \#(16)$$

$$1.5\%\ Quatsome\ SAM \rightarrow d =\ 2\sqrt{\frac{1}{\pi\cdot\left(1.22\,\frac{molecules}{nm^2}\cdot 0.015\right)}} = 8.34\ nm\ \#(17)$$

$$0.125\%\ Quatsome\ SAM \rightarrow d =\ 2\sqrt{\frac{1}{\pi\cdot\left(1.22\,\frac{molecules}{nm^2}\cdot 0.00125\right)}} = 28.89\ nm\ \#(18)$$

As seen in the previous estimations, if Quatsomes and filler PEG molecules were to distribute on the surface of the SAM according to the molar ratios, the surfaces would probably be saturated with Quatsomes in all cases. This is due to the average radius of

Quatsomes of approximately 35 nm being higher than the estimated Quatsome to Quatsome distance in all cases.

As such, the estimated bulk RGD density in these surfaces would be close to that of the own Quatsome membrane, even though with increasing proportions of PEG-SH in the mixture a lower incorporation of Quatsomes to the SAM is to be expected.

Interestingly, the results showed on the main text for both biological and impedimetric assays hint at a similarity in structure between the SAM RGD-QS 10% and the SAM RGD-QS 1.5% surfaces, while showing much different results for the SAM RGD-QS 0.125%.

Additionally, RGD-RGD distances in hybrid SAMs are defined by the average RGD surface density found on the Quatsome membrane. The same calculation can be made:

$$d(RGD - QS\ 100\%) = 2 \cdot \sqrt{\frac{1}{0.078\pi}} = 4.04\ nm\ \#(19)$$

Table S1. Summary of the estimated bulk and local average surface RGD densities and average RGD-RGD distance across all the prepared substrates. SAM RGD-QS X% are the QS-based hybrid SAMs that feature a mixture of RGD-QS-PEG_{3000}-SH and PEG_{130}-SH, with the X% featuring the ratio of Quatsomes over PEG filler molecules. SAM RGD X% are the mixed SAMs that feature a mixture of HS-$(CH_2)_8$-PEG_{130}-OH and Cys_3-PEG_{260}-c(RGDfE) molecules, with the X% featuring the ratio of RGD-presenting molecules over PEG filler molecules.

**Platform**	**Sample**	**Estimated maximum bulk average surface RGD density (RGD units/nm^2)**	**Estimated maximum local average surface RGD density (RGD units/nm^2)**	**Estimated RGD-RGD distance (nm^2)**
Mixed SAM RGD	SAM PEG 100%	0	0	-
	SAM RGD 100%	1.22	1.22	1.02
	SAM RGD 10%	0.122	0.122	3.25

	SAM RGD 1%	0.0122	0.0122	11.28
Hybrid SAM RGD-QS	SAM BLANK-QS 100%	0	0	-
	SAM RGD-QS 100%	0.078	0.078	4.04
	SAM RGD-QS 10%	~0.0078-0.078	0.078	4.04
	SAM RGD-QS 1.5%	~0.0012-0.078	0.078	4.04
	SAM RGD-QS 0.125%	~0.0001-0.078	0.078	4.04

5. Young's modulus of quatsomes in quasi-suspension

The Young's modulus of SAM RGD-QS 0.125% (with PEG130-SH filler) was obtained from QS indentation curves obtained by AFM.

For small vesicle deformations (smaller than the membrane thickness), the vesicle indentation increases approximately linearly with the applied force (Fig. SI7), the slope representing the vesicle membrane stiffness (k_{mem}).

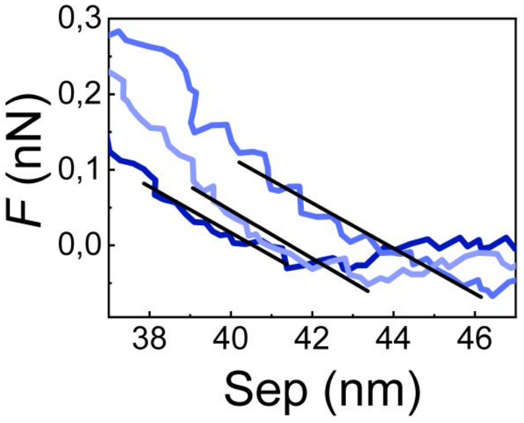

Figure S7 Force vs. tip-sample separation approach curves showing QS indentation for SAM RGD-QS 0.125% (with PEG130-SH filler), and corresponding linear fits to the initial indentation, from which the membrane stiffness is obtained.

The effective Young's modulus E can be obtained through the following equation,[4]

$$E = \frac{k_{mem} \cdot R\sqrt{3(1-\nu^2)}}{4 \cdot h^2} \#(20)$$

with *R* being the vesicle radius, *h* the membrane thickness, and ν the Poisson ratio. The latter is assumed as 0.5 in this case, the typical value employed for lipid vesicles.[5] The vesicle radius is obtained from the tip-sample separation at which vesicle indentation sets in, and the membrane thickness from membrane rupture events (Fig. 8B).

To obtain a rough estimate of the order of magnitude of the Young's modulus, we have analyzed the indentation curves of three vesicles with representative diameter values:

Table S2. Vesicle diameter, membrane stiffness, membrane thickness and Young's modulus of three vesicles analyzed from indentation curves under AFM.

**Vesicle diameter (nm)**	46	43	40
**Membrane stiffness (N/m)**	0.03	0.032	0.03
**Membrane thickness (nm)**	4.9	4.9	5
**Young's modulus (MPa)**	11	11	10

For these quatsomes, which are in quasi-suspension, we obtain a Young's modulus of 10.7 ± 0.5 MPa.

6. References

(1) Conway, J. H.; Sloane, N. J. A. *Sphere Packings, Lattices and Groups*, Second Edi.; Springer Science+Business Media, LLC, **2013**. https://doi.org/10.1007/978-1-4757-2249-9.

(2) Ferrer-Tasies, L.; Moreno-Calvo, E.; Cano-Sarabia, M.; Aguilella-Arzo, M.; Angelova, A.; Lesieur, S.; Ricart, S.; Faraudo, J.; Ventosa, N.; Veciana, J. Quatsomes: Vesicles Formed by Self-Assembly of Sterols and Quaternary Ammonium Surfactants. *Langmuir* **2013**, *29*

(22), 6519–6528. https://doi.org/10.1021/la4003803.

(3) Vargas-Nadal, G.; Muñoz-Ubeda, M.; Alamo, P.; Arnal, M. M.; Céspedes, V.; Köber, M.; Gonzalez, E.; Ferrer-Tasies, L.; Vinardell, M. P.; Mangues, R.; Veciana, J.; Ventosa, N. MKC-Quatsomes: A Stable Nanovesicle Platform for Bio-Imaging and Drug-Delivery Applications. *Nanomedicine Nanotechnology, Biol. Med.* **2020**, *24*, 102136. https://doi.org/10.1016/j.nano.2019.102136.

(4) Delorme, N.; Fery, A. Direct Method to Study Membrane Rigidity of Small Vesicles Based on Atomic Force Microscope Force Spectroscopy. *Phys. Rev. E* **2006**, *74* (3), 030901. https://doi.org/10.1103/PhysRevE.74.030901.

(5) Laney, D. E.; Garcia, R. A.; Parsons, S. M.; Hansma, H. G. Changes in the Elastic Properties of Cholinergic Synaptic Vesicles as Measured by Atomic Force Microscopy. *Biophys. J.* **1997**, *72* (2), 806–813. https://doi.org/10.1016/S0006-3495(97)78714-9.
